# Development, validation and a GAPI greenness assessment for the determination of 103 pesticides in mango fruit drink using LC-MS/MS

**DOI:** 10.3389/fchem.2023.1283895

**Published:** 2023-11-21

**Authors:** Madhu Tippannanavar, Tirthankar Banerjee, Sumit Shekhar, Sudama Ram Sahu, Bijendra Singh, Neethu Narayanan, Shalini Gaur Rudra, Bidisha Chakrabarti, Suman Gupta, Anupama Singh

**Affiliations:** ^1^ Division of Agricultural Chemicals, New Delhi, India; ^2^ The Graduate School, New Delhi, India; ^3^ Division of Food Science & Post Harvest Technology, New Delhi, India; ^4^ Division of Environmental Science ICAR Indian Agricultural Research Institute, New Delhi, India

**Keywords:** LC-MS/MS, pesticides, QuEChERS, processed foods, mango fruit drink

## Abstract

A robust method was developed using LC-ESI-MS/MS-based identification and quantification of 103 fortified pesticides in a mango fruit drink. Variations in QuEChERS extraction (without buffer, citrate, and/or acetate buffered) coupled with dispersive clean-up combinations were evaluated. Results showed 5 mL dilution and citrate buffered QuEChERS extraction with anhydrous (anhy) MgSO_4_ clean-up gave acceptable recovery for 100 pesticides @ 1 μg mL^−1^ fortification. The method was validated as per SANTE guidelines (SANTE/11813/2021). 95, 91, and 77 pesticides were satisfactorily recovered at 0.1, 0.05, and 0.01 μg mL^−1^ fortification with HorRat values ranging from 0.2–0.8 for the majority. The method showed matrix enhancement for 77 pesticides with a global uncertainty of 4.72%–23.89%. The reliability of the method was confirmed by real sample analysis of different brands of mango drinks available in the market. The greenness assessment by GAPI (Green Analytical Procedure Index) indicated the method was much greener than other contemporary methods.

## Graphical Abstract

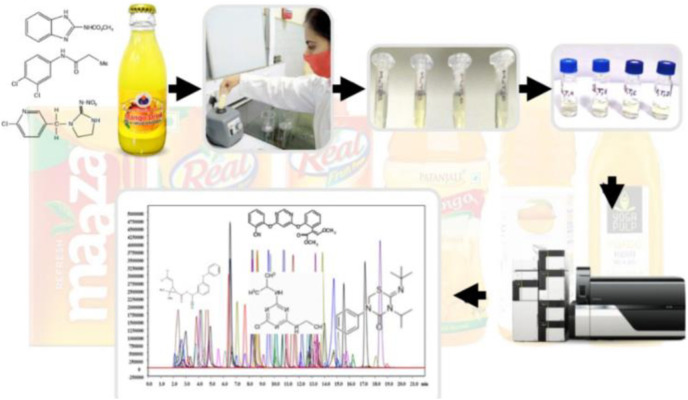

## Highlights

• Multi residue LC-MS/MS method for the detection and quantification of 103 pesticides in mango fruit drink
• QuEChERS method and dilution volume optimization for the effective extraction and clean-up of fortified pesticides
• 5 mL dilution, citrate QuEChERS extraction, and anhy MgSO_4_ clean-up gave acceptable recovery (70%–120%) for 100 pesticides with <20% RSD
• Method validation and real sample analysis
• GAPI tool-based greenness assessment

## 1 Introduction

Mango (*Mangifera indica*), the king of Indian fruits and a member of the Anacardiaceae family, is one of the most significant and commonly grown fruits in India and other tropical nations. A rich profile of vitamins and minerals, good amounts of carbs, proteins, fats, and dietary fiber make mango a nutrient-dense and satiating choice for a balanced diet. It is a rich source of a plethora of phytochemicals like quercetin, isoquercitrin, astragalin, fisetin, gallic acid, and abundant enzymes (Siddiq et al., 2017).

Considering its aesthetic values, strong aroma, delicious taste, high nutritive values, and antioxidant properties, the fruit is served as whole fruit, fruit juice, smoothies, ice cream, chutney, etc., and highly impacts on domestic and international trade. The most popular and globally consumed product of processed mango is mango fruit drink. Mangoes are infested by many pests thus vastly affecting the trade (Pena et al., 1998). To manage the losses by pests and diseases, numerous pesticides of different classes like insecticides and plant growth regulators are in use on mango (CIBRC, 2022). But, their unscientific use in agriculture has engraved the problem of residues in mango fruits (Mukherjee et al., 2007).

Consequently, mangoes are no longer regarded as the king of tropical fruits in much of Europe; instead, they are now considered to be a prohibited fruit based on the fact that 207 consignments were returned by the European Union (EU) in 2014 (Business standards, 2014). With technological upliftment and increased socio-economic status of the people, food safety concerns in terms of pesticide residues are nowadays attaining wide focus (Nougadere et al., 2020). Therefore, it is crucial to keep an eye on pesticide residues in processed products like mango fruit drinks, especially in light of their consumption by the most vulnerable section of society, i.e., infants, children, and old and infirm persons, for whom any detectable pesticide residue raises the question about safety.

The low concentration of analytes and the abundance of additives and interfering compounds that might be coextracted with analytes pose a challenge in detecting pesticide residues in food matrices, which in most cases negatively impacts the analytical results (Wilkowska and Biziuk, 2011; Tang et al., 2004 used liquid–liquid extraction followed by SPE for clean-up and GC analysis for quantifying four pyrethroid pesticides in apple juices. Zang et al., in 2014 used the QuEChERS–DLLME method for fruit juices of complex matrices (orange, lemon, kiwi, and mango) and found its suitability for the quantification of 10 pyrethroid insecticides. Rizzetti et al., in 2016 had developed a UHPLC–MS/MS method for multi-residue determination of 74 pesticides in orange juice. Sivaperumal et al. (2017) used the UHPLC-Q-TOF/MS method for the determination of 68 pesticides in the mango fruit matrix. A UHPLC-MS/MS method was developed for quantification of 113 pesticides in green and ripe mangoes by Li et al., in 2018.

However, methods for multi-residue pesticide analysis in processed foods are scant in number, and in the case of mango fruit drink, the Multiple reaction monitoring for most of the most commonly used pesticides in the Indian Scenario is not available so far. Zambonin et al. (2004) demonstrated varied recoveries for eight organophosphorus pesticides (diazinon, ethyl-parathion, fenitrothion, fenthion, malathion, methyl-parathion, methidathion, and phorate) in orange, grapefruit, and lemon due to significant variation in sample matrices, even though all three samples represent citrus fruits and belong to the Rutaceae family. In the multi-residue study for 22 GC-amenable and 21 LC-amenable pesticides made by Damale et al. (2023) using GC-MS/MS and LC-MS/MS on four different Indian pomegranate cultivars, resulted in a unique matrix effect and thus acute variation for each pesticide. Sarkar et al. (2022) also identified huge variations in the composition of citrus fruits (kinnow, mosambi, and orange) for phenolic compounds, flavonoids, and antioxidant potency.

Therefore a method solely for mango fruit drink is needed for the identification and quantification of multi-residues with utmost importance.

Hence, in this study, QuEChERS-based d-SPE extraction-cum-clean-up coupled with advanced liquid chromatography tandem mass spectroscopy (UPLC-MS/MS) method has been developed for trace level determination of 103 pesticides in the mango fruit drink matrix. The approach offers excellent selectivity, high sensitivity, and a broad range of applications for the determination of multiple residues in mango fruit drinks. The evaluation of 103 pesticide residues in mango fruit drinks prevailing in the local market was also performed using the suggested approach.

To evaluate the greenness of the developed method, the GAPI green chemistry tool was employed in the study starting from sample collection, extraction, and cleanup to final determination by the instrument.

## 2 Materials and methods

### 2.1 Standards

Sigma-Aldrich Chemie GmbH, Germany provided Certified Reference Materials (CRM) for 103 regularly used pesticides in the Indian context, including acaricides, fungicides, herbicides, insecticides, plant growth regulators, and rodenticides. A list of the pesticides and their intended purpose, molecular weight, purity percentage of CRM, and MRL of pesticides recommended in mango are listed in Supplementary Table S1.

### 2.2 Chemicals, solvents, and apparatus

Ammonium formate, NH_4_HCO_2_ [98% pure], was obtained from Sisco Research Laboratories Pvt. Ld., Mumbai, India. Anhydrous magnesium sulphate of >98% purity (used after heating at 600°C for 6 h for removal of phthalates and traces of moisture) employed in the extraction process was procured from Thermo Fisher Scientific, India. Anhydrous sodium chloride of AR Grade (Merck, India), used for extraction, was pre-washed with acetone and activated at 600°C for 6 h in a muffle furnace before use. Salts like trisodium citrate dehydrate [Na_3_C_6_H_5_O_7._2H_2_O] (98%, AR Grade Molychem, India) and disodium hydrogen citrate [Na_2_C_6_H_6_O_7._1.5H_2_O] (99.8% pure, AR Grade, Molychem, India) were used for QuEChERS extraction. Primary Secondary Amine (PSA) of size 40 µm size, and Octadecyl modified silica (C18) of 57.5 µm size were procured from Agilent Technologies (Santa Clara, CA) were used for QuEChERS clean-up. Acetone (minimum 99.8% pure, HPLC grade (MERCK, India) is used for cleaning purposes. Acetonitrile (Hypergrade for LC-MS, Merck LiChrosolv) was used for pesticide residue extraction. Methanol (Gradient grade for liquid chromatography, Merck LiChrosolv) was used in mobile phase during instrumental analysis. Water-having a resistivity of 18.2 MΩ cm @ 25°C was obtained from the Millipore water purification system (Milli-Q, Academic, Millipore, United States) and used in sample preparation and in the mobile phase as well. A-grade 10 mL volumetric flask (Borosil^®^, India), analytical Balance [0.1 g–220 g, sensitivity 0.1 mg] (METTLER, Switzerland TOLEDO ME-204), calibrated Micropipette of 0.1–1 mL (Thermo Scientific, Germany), calibrated Micropipette of 2–20 µL (Sartorius, United States), calibrated Micropipette of 0.5–5 mL (Eppendorf, United States), Oakridge centrifuge tube (50 mL, Tarsons, India), vortex mixer (Model Spinix, Tarson, India), sample filtration syringe (Hamilton, Gastight^®^ #1005, 5 mL capacity), syringe filter (Qualisi/Nylon Syringe Filter 13 mm*0.22 ȕm), vials (2 mL clear Screw cap vials, Thermo Fisher Scientific, India) were employed for sample preparation.

### 2.3 Preparation of standard stock solution

A primary stock solution of 1,000 μg mL^−1^ concentration for each pesticide was prepared in acetonitrile in an A-grade 10 mL volumetric flask (Borosil^®^, India). An intermediate standard mixture of 103 pesticides of conc. 100 and 10 μg mL^−1^ and their working solutions of lower concentrations (1, 0.5, 0.1, 0.05, 0.01, 0.005, and 0.001 μg mL^−1^) were prepared from primary stock solution by serial dilution technique and volume made up using acetonitrile.

### 2.4 Spiking of mango fruit drink with pesticides and sample processing

A 200 mL Mango fruit drink (Pusa Mango drink) prepared by using organically grown pesticide-free mangoes, was procured from the Division of Post-Harvest Technology, IACR-IARI, New-Delhi, 110012.

Mango fruit drink prepared as per recommended procedure (Sethi et al., 2006) from organically grown mango, was taken in a 50 mL Oakridge centrifuge tube and added with a standard mixture of 103 pesticides to attain the desired fortification level. After shaking the tube, the material was kept for 2 h in ambient condition (27°C ± 1°C), subsequently homogenized using a hand-held homogenizer, and placed in an ultrasonic bath for 5 minutes before extraction.

**FIGURE 1:**
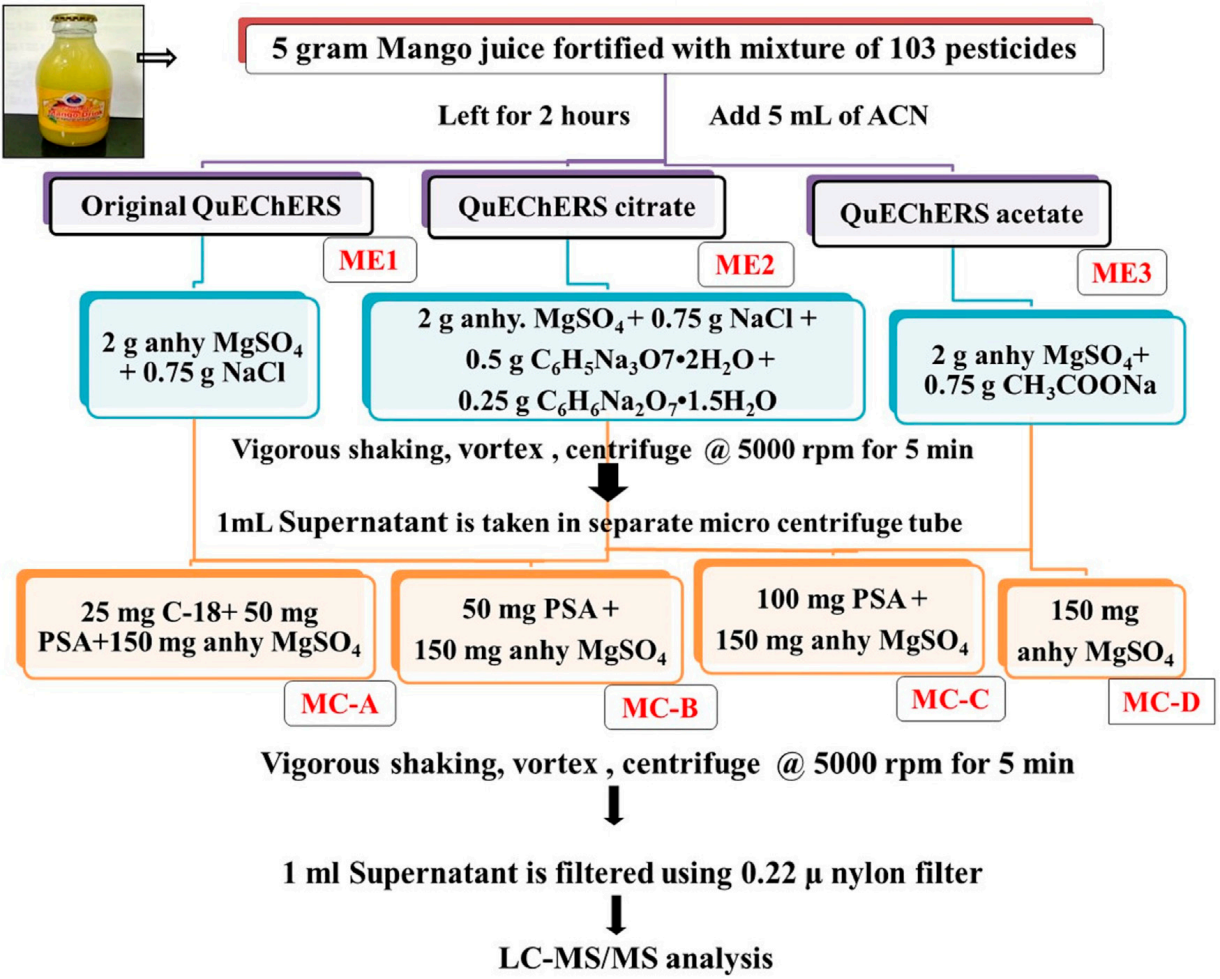
Flow diagram of optimization of modified QuEChERS extraction and cleanup methods in mango fruit drink.

Optimization of sample preparation by QuEChERS extraction (original QuEChERS, modified buffered QuEChERS using citrate and acetate buffers) and clean-up procedures (using combinations of anhydrous MgSO_4_, PSA, C-18) were tried and are depicted in Figure 1. Once the QuEChERS extraction method is optimized, the effect of dilution on extraction/clean-up performance using varied combinations of clean-up agents was evaluated by diluting the mango drink at different levels (0, 2, 4, 5 mL) using milli Q water prior to extraction.

### 2.5 Liquid Chromatography-⁻Tandem mass spectroscopy (LC–MS/MS) and method development

Quantification of the target pesticides was done using Shimadzu LC-MS/MS-8030 (UHPLC model-Nexera, LC-30AD Liquid Chromatography, SIL- 30AC auto-injector (Shimadzu Corporation, Kyoto, Japan) coupled with Triple Quadrupole Mass Detector. Zorbax Eclipse Plus C-18 column, 3 mm i. d., 10 cm length with 3.5 µm column particle size (Agilent Technologies, United States make) column was used. Optimization of LC-MS/MS parameters is a prerequisite to identifying and quantifying the residues of multiclass pesticides. In LC, the mobile phase was a mixture of A (80:20 5 mM ammonium formate buffer dissolved in water: methanol) and B (10: 90 5 mM ammonium formate buffer dissolved in water: methanol) used at a flow rate of 0.2 mL min^−1^ under gradient programming for 22 min runtime. Initially, mobile phases A and B were used in 45% and 55% proportion respectively for 1 min and gradually increased to 100% of mobile phase B within 13 min and maintained until 16.5 min. After 16.5 min, they were brought to the initial proportion of 45% (A) and 55% (B) and maintained until 22 min. A 2 μL sample volume was injected in each run. The Mass Spectrophotometer was operated under Electrospray Ionization (DUIS–ESI interface) in both positive and negative modes for optimization of unique multiple reaction monitoring (MRM) transitions for each pesticide separately. Nitrogen was used as nebulizing gas and drying gas at 3.0 L min^−1^ and 15 L min^−1^ flow rates respectively. Ultrapure Argon was used as Collision-induced dissociation (CAD) gas. Desolvation line temperature (DL) and heat block temperatures were maintained at 120°C and 300°C respectively. For each pesticide, retention time, Q1 pre-bias, Q3 pre-bias, and collision energy were optimized individually and are mentioned in Supplementary Table S1. Software Lab Solutions Version 5.86, was exercised in data acquisition and analysis.

### 2.6 Single laboratory validation of the developed method

The suitability and applicability of the developed multi residues analysis method were assessed by single laboratory validation as per the SANTE guidelines (2021). The parameters considered as per the guidelines were linearity, specificity, limit of detection (LOD), limit of quantification (LOQ), accuracy, precession, and uncertainty measurement.

#### 2.6.1 Linearity

The calibration curve (concentration-response) for a mixture of 103 pesticides injected under optimized method parameters was accomplished using 7 different concentration levels of 0.001, 0.005, 0.01, 0.05, 0.1, 0.5, and 1 μg mL^−1^. Correlation coefficients and regression equations for all the pesticides are given in Supplementary Table S1.

#### 2.6.2 Specificity

To achieve the specificity of identification, the reagent blank was compared with the fortified sample. Detection of the target greater than the detection limit is considered to be the specificity criterion (Banerjee et al., 2019).

#### 2.6.3 Sensitivity

The sensitivity of the developed method was measured in terms of the detection limit (LOD) and the quantification limit (LOQ) for 103 pesticides in a mango fruit drink. Method LOD was obtained by spiking the blank sample at different fortification levels. LOD and LOQ are considered the concentrations at which the S/N (signal-to-noise ratio) are ≥3/1 and ≥10/1, respectively (Banerjeee et al., 2019). LOQ was based on pre-determined acceptance criteria of 70%–120% recovery and ≤20% RSD. At each analysis, the signal-to-noise ratio of the quantifier transition peak was calculated using the Lab Solution software.

#### 2.6.4 Accuracy

Accuracy in terms of recovery was studied in triplicates at 0.01, 0.5, 0.1, and 1 μg mL^−1^. Recoveries lying between 70% and 120% were considered acceptable recoveries as per SANTE 2021 guidelines. Recoveries of the fortified pesticides in mango fruit drink were calculated against solvent standard (standard solution prepared in acetonitrile) (Eq. 1) as well as in matrix-matched standard (prepared through post-extraction spiking of blank samples) (Eq. 2) and corrected recoveries were determined as per following equations.

$$\%\text{ Recovery}{\;}_{\left(\text{against}\;\text{solvent}\;\text{standard}\right)}\;\left[\text{RSS}\right]=\left(\frac{Peak\;area\;of\;the\;spiked\;sample}{Peak\;area\;of\;the\;solvent\;standard}\right)x\;100$$ (1)

$$\%\text{ Recovery}{\;}_{\left(\text{against}\;\text{matrix}\;\text{match}\;\text{standards}\right)}\;\left[\text{RMM}\right]=\left(\frac{Peak\;area\;of\;the\;pre-extraction\;spiked\;sample}{Peak\;area\;of\;the\;post-extraction\;spiked\;sample}\right)x\;100$$ (2)

Where, Recovery <70% = not acceptable, 70%–120% = acceptable, >120% = not acceptable.

#### 2.6.5 Precession- repeatability

The precession of the protocol was confirmed in terms of intra-laboratory repeatability, which was assessed independently at each level of fortification (0.1, 0.05, and 0.01 µg mL^−1^) using the Horwitz ratio (HorRat) (Horwitz and Albert, 2006). The ratio (Eq. 3) is determined for each pesticide to determine whether the procedure is acceptable or not in terms of precision.

HorRat=RSD/Prsd (3)

Where, RSD stands for relative standard deviation and Prsd is predicted relative standard deviation, which is computed using the formula Prsd = 2C^−0.15^, where C is the mass fraction of the concentration (1 ng/mL = 1 × 10^−9^). The analytical approach is unquestionably suspected to perform worse than expected if the HorRat is more than 1; if the HorRat is <<1, it is suspected that the collaborative trial was improperly conducted and produced overly optimistic precision values; and if the HorRat is between 0.3 and 1, the method precision in terms of reproducibility is close to the predicted value.

0.3≤ HorRat ≤1 Totally acceptable recommended range

HorRat<0.3 or 1< HorRat≤2 Acceptable but reasonable explanation is required

HorRat>2 Not Acceptable

#### 2.6.6 Estimation of uncertainty

A fishbone diagram was created for potential contributors to the uncertainty after the potential causes of uncertainty were defined at the outset (Supplementary Figure S1). For all 103 pesticides in mango fruit drink, the uncertainty related to purity of CRM (Uc), analytical balances (Um), volumetric flask (Uf), micropipettes (Ug and Uh) (Ud and Ue), recovery (Ub) and instrument (Ua) results were assessed in terms of combined or total standard uncertainty and subsequently as extended or global uncertainty (Eq. 5). (Banerjee et al., 2019).

The global uncertainty was determined as shown below.

$$GU=\sqrt{\left(U_{S1}^{2}+U_{S2}^{2}+U_{S3}^{2}+U_{r1}^{2}+U_{r2}^{2}+U_{r3}^{2}+\ldots \right)}$$

$$U_{e}=k\times U_{c}$$

$$Combined\;or\;total\;uncertainty\;\left(\%\right)=\frac{U_{e}\times 100}{Concentration\;@\;LOQ}$$ (4)

Where, GU is global uncertainty and U_e_ is expanded uncertainty, and k is coverage factor 2.

### 2.7 Matrix effect

The matrix effect is represented as peak enhancement (+ve) and suppression (−ve) and was studied by comparing calibration curves prepared in solvent (solvent standard) and in blank (matrix-matched standard) as per IUPAC technical report (Thompson et al., 2002; Banerjee et al., 2007; Jadhav et al., 2017; Shinde et al., 2021). The matrix effect was calculated using the following formula,

$$\text{Matrix Effect }\left(\text{ME }\%\right)=\frac{\left(Peak\;area\;of\;matrix\;matched\;standard-Peak\;area\;of\;the\;solvent\;standard\right)\times 100}{Peak\;area\;of\;the\;solvent\;standard}$$ (5)

If ME is positive (+), matrix enhancement and negative (−) means, matrix suppression.

### 2.8 Method validation in real samples

To validate, the recommended multi-residue approach was used to quantify any residues that might have been present in real-market mango fruit drink samples from 10 different brands or firms that were bought and kept in their original packaging until analysis. Utilising the newly developed modified QuEChERS (citrate) method, extraction and cleanup were carried out, and LC-MS/MS analysis was performed.

### 2.9 Assessment of the developed method as per green chemistry

**FIGURE 2:**
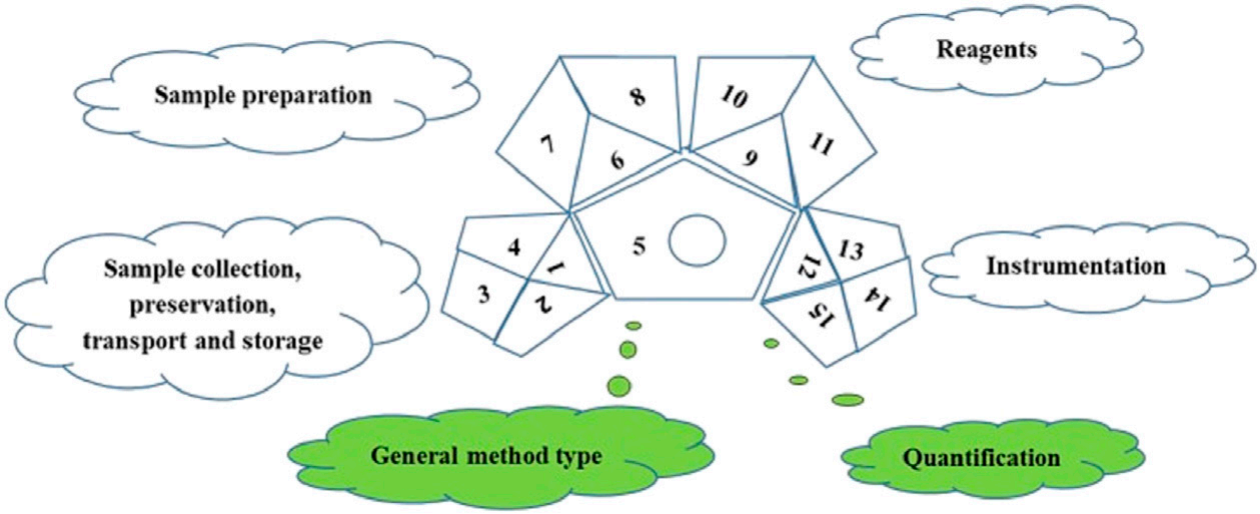
Green Analytical Procedure Index pictogram with description (Płotka-Wasylka, 2018).

Analytical methods with a green perspective, Multiple reaction monitoring (MRM) are being developed to help a variety of analytes be recognised in a single analytical run. The challenge, however, is that the molecules that must be identified are present at very low concentrations and have various physical and chemical properties based on their chemical makeup. One new idea in sustainable development is “green analytical chemistry.” As a result, the recently evolving analytical techniques ought to satisfy the requirements of green chemistry. The green analytical techniques are made to use safe ingredients, consume as little energy as possible, and produce as little waste as possible while still being effective. As a result, the goal of most analytical techniques is to use environmentally friendly solvents and a smaller, more straightforward sample preparation stage (Soltani and Sereshti, 2022). To evaluate the greenness of the study, a unique Green Analytical Procedure Index (GAPI) tool was employed. GAPI is a semi-quantitative tool consisting of five pentagrams representing 1) sampling process, 2) sample preparation, 3) reagents and chemicals, 4) instrumentation, and 5) the general method, which provides sufficient data to assess and measure the environmental impact associated with each step of an analytical approach from sampling through the final instrumental analysis. The three major colours of the symbol—green, yellow, and red—denote low, medium, and high impact, respectively (Płotka-Wasylka, 2018) and provides sufficient data to evaluate the greenness of an entire analytical process, from sampling through the final instrumental analysis. The analytical process in GAPI comprises five primary steps:

In the green assessment, 15 parameters were considered (Figure 2) and the greenness of the developed (M.IV.) multi-residue LC-MS/MS method for 103 pesticides in mango fruit drink was compared with three other existing methods (M.I. and M. II. and M. III.) in mango drinks for the residue/multi-residue analysis.

M.I. = Naz et al., Application of High-Performance Liquid Chromatography to the Analysis of Pesticides in mango juice.

M.II. = Zhang et al., 2014. Determination of ten pyrethroids in various fruit juices: Comparison of dispersive liquid–liquid microextraction sample preparation and QuEChERS method combined with dispersive liquid–liquid microextraction.

M.III. = Deme and Upadhyayula, 2015. Ultra-performance liquid chromatography atmospheric pressure photoionization high-resolution mass spectrometric method for determination of multiclass pesticide residues in grape juice and mango juice.

M.IV. = Development, validation, and a GAPI greenness assessment of LC-MS/MS-based method for analysis of 103 pesticides in mango fruit drink (Developed method).

## 3 Results and discussion

### 3.1 Optimization of LC–MS/MS system

For the identification and quantification of 103 pesticides, the instrumental method was optimized using Ultra Performance Liquid Chromatography-tandem Mass Spectroscopy [Shimadzu LC-MS/MS-8030]. For the ionization, electron spray ionization operating in both positive and negative modes were employed. Method optimization was done by sequential molecular ion scan for the selection of the most abundant precursor ion and it was isolated in the first quadrupole. Different collision energies were optimized to obtain corresponding product ions and thus optimized the MRM transitions (Supplementary Table S1). ESI (+) ionization achieved the best results for most of the pesticides, while pesticides like bentazone, fipronil, flubendiamide, metaflumizone, and propanil exhibited higher abundance in ESI (−) mode. Cabrera et al., 2016 observed that ESI (−) gave better results for bentazone, fipronil, metsulfuron-methyl, and pyrazosulfuron ethyl where acetic acid was used as a mobile phase modifier. Gradient programming of the mobile phase ensured the separation of multi-class pesticides with different polarities. By gradually increasing the proportion of mobile phase B (10:90, water: methanol) to 100%, most of the polar pesticides like neonicotinoids [dinotefuron (2.49 min), thiamethoxam (2.77 min), imidacloprid (3.14)], sulphonyl urea herbicides [metsulfuron methyl (2.67 min), azim sulfuron (3.44)], oxithiin carbaxamides [oxicarboxin (4.12), carbaxin (9.24)], and triazines [tricyclazole (4.72 min), myclobutanil (10.85)], were eluted early in less than 13 min. Medium polar pesticides viz., many synthetic pyrethroids [alpha-cypermethrin (14.90), bifenthrin and tetramehtrin (15.22), cyhalothrin lamda (16.79)], alinides [pretilachlor (14.67), butachlor (14.93)], dinitroanilines [pendimethalin (16.21), isopropalin (16.90)], [phenoxy acid ester herbicides [haloxyfop-methyl (14.07), cyhalofop-butyl (14.14), diclofop-methyl (15.17)], and strobilurins [azoxystrobin (10.02 min)] were eluted between 13 min and 17 min, where 100% of mobile phase B is maintained. The nonpolar or sparingly soluble pesticides like quinazolines [fenazaquin (18.38)], triazine [bitertenol (19.21 min)], etc. Were eluted after 17 min with a mobile phase mixture of 45% of A and 55% of B. Use of methanol in higher percentage for improving the separation, also improved the sensitivity (both ESI +/−) for many of the phenoxy acid and OP pesticides.

**FIGURE 3:**
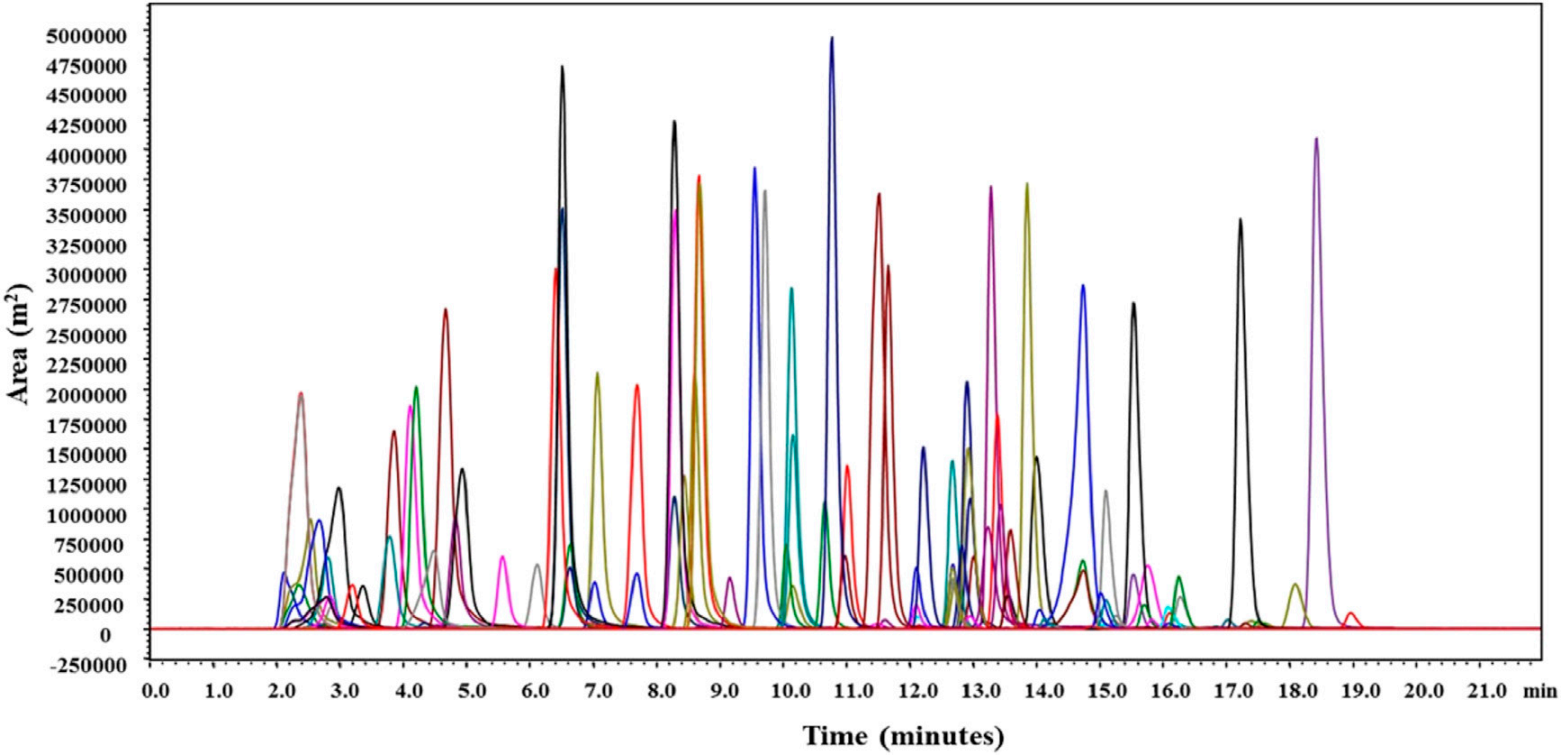
Total ion chromatograms of detected pesticides.

To improve analyte signals, to obtain better reproducibility and chromatographic responses, 5 mM Ammonium formate buffer was used as a mobile phase modifier. Ammonium ions formed from ammonium formate buffer supress the sodium adducts formation during ionization, which wase quite common under acidic conditions. Thus, pesticides predominantly form [M + H]^+^ for most of the pesticides and [M + NH_4_]^+^ molecular ions were formed by most of the synthetic pyrethroids (alpha-cypermethrim, bifenthrin, cyhalothirn-lamda, cyphenothrin, fenvelarate, flucythrinate, permetrin), carfentazone ethyl, cyhalofop-butyl, diclofop-methyl, lactofen. Similar results were noticed by (Hiemstra and de Kok, 2007; Riedel et al., 2010; Stotcheva, 2011) where pyrethroids, diclofop-methyl, etc. have shown much higher sensitivity, better reproducibility, and response due to [M + NH_4_]^+^ ionization when mobile phase buffers like ammonium formates or acetates were used. The above-mentioned method’s optimised LC-MS/MS conditions provided excellent separation for the target analytes, 100 pesticides in the mango fruit drink. Pesticides along with their retention time during elution are given in Supplementary Table S1. Total ion chromatograms in overlay and their retention time of all detected pesticides are presented in Figure 3.

### 3.2 Investigation of the QuEChERS method

**FIGURE 4:**
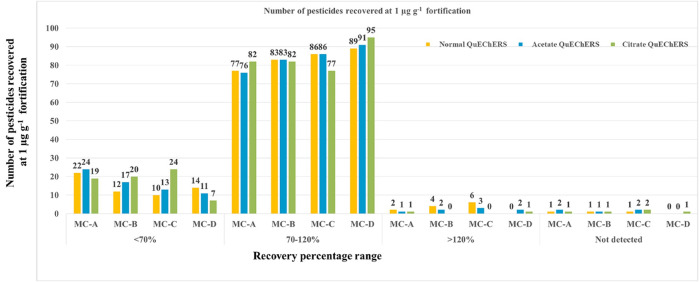
Recovery of pesticides by different QuEChERS extraction and cleanup combinations.

Antioxidant compounds present in mango affect the pesticidal extraction and quantification. Hence three varied QuEChERS extraction methods and four clean-up combinations were tried to extract 103 pesticides and ensure their selective quantification in the presence of undesirable interfering matrix components. The QuEChERS method uses fewer solvents and reagents during sample preparation/extraction-cleanup, thus helping to improve ecological integrity, hence QuEChERS methods were exploited in this study. Acetonitrile was used as an extraction solvent. Original QuEChERS (ME1), citrate buffered QuEChERS (ME2), and acetate buffered (ME3) extraction methods were tried along with various combinations of clean-up agents like 25 mg C-18 + 50 mg PSA +150 mg anhy. MgSO_4_ (MC-A), 50 mg PSA +150 mg anhy. MgSO_4_ (MC-B), 100 mg PSA +150 mg anhy. MgSO_4_ (MC-C) and only 150 mg anhy. MgSO_4_ (MC-D) [Figure 1]. Among all these combinations, buffered citrate QuEChERS extraction (ME2) carried out using 2 g anhydrous magnesium sulphate (MgSO_4_), 0.75 g sodium chloride (NaCl), 0.5 g trisodium citrate dehydrate [Na_3_C_6_H_5_O_7._2H_2_O], disodium hydrogen citrate [Na_2_C_6_H_6_O_7._1.5H_2_O] and in most of the clean-up combinations gave acceptable recovery (70%–120%) for most of the pesticides. A number of recovered pesticides using all three QuEChERS methods are given in Figure 4 and the recovery percentage of all the pesticides is given in Supplementary Table S1. With the use of citrate buffers, the pH of the extract rose to 5.29 from 4.05 (pH of juice) thus facilitating the extraction of low pH sensitive pesticides more efficiently by improving the selectivity from the co-extractives, which yielded good recoveries for most of the acidic pesticides like alpha-cypermethrin, flucythrinate, etc. Similar results were observed by Prestes et al. (2009) where they used acetate and citrate buffers to extract low-pH susceptible compounds, such as thiabendazole and imazalil from the food matrix.

Since mango fruit drink typically contains 80%–95% water, and separation of the analyte from water is a critical step in extraction. Acetonitrile, as an extracting solvent, provides extraction of a wide range of pesticides with variable polarities, and it can be easily separated from water. Once the QuEChERS extraction method was optimized, the effect of dilution using mili Q water at varied levels (0, 2, 4, 5 mL) and four clean-up combinations were studied for a maximum number of pesticidal recovery (Supplementary Table S3).

**FIGURE 5:**
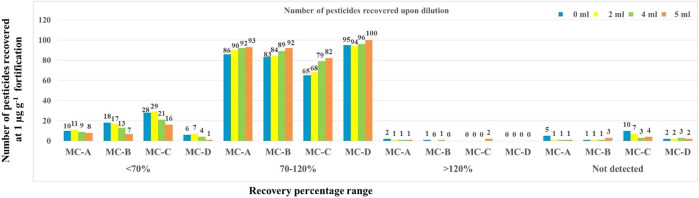
Effect of dilution in Citrate QuEChERS extraction and cleanup technique on recovery of pesticides.

In ME2-MC-A, ME2-MC-B, ME2-MC-C, and ME2-MC-D, the effect of dilution had a considerable impact on acceptable recovery. With the increase in dilution volume from 0 mL to 5 mL, the number of pesticides recovered was also increased in all the combinations (Figure 5). At 5 mL dilution, the treatment combination ME2-MC-D recovered the highest number of pesticides (100) in the acceptable range with <20% RSD compared to all other treatment combinations.

Though mango fruit drink has sufficient water, the presence of antioxidants, sugar, and other compounds present in mango and preservatives used in fruit drink might get in the way of extraction the and instrumental identification and quantification of pesticides. Hence, dilution of this drink prior to extraction was effective in reducing the interfering matrix components. In the LC-MS/MS method, optimization using unique mass by weight-based quantifier and qualifier MRM transitions for each pesticide ensured the targeted detection and quantification in an acceptable range even in the diluted sample. Hence, 5 mL dilution and ME2-MC-D (anhy MgSO_4_ alone as clean-up agent) was considered best for the maximum number of pesticides.

Since anhy MgSO_4_ was used alone in this clean-up treatment, it has given a good amount of acceptable recovery for the highest number of pesticides. Anhy MgSO_4,_ has not adsorbed any pesticides onto it thus ensuring good clean-up and recovered the maximum number of pesticides. The RSD of most of the pesticides was less than 20%, which shows the good precession of the method. Anhy MgSO_4_ when used in extraction, increased the ionic strength of the aqueous mixture and helped in binding large amounts of water. It also absorbed traces of water left in the clean-up step. Sodium chloride in extraction helped in increasing the ionic strength of the aqueous phase and also aided in phase separation.

In d-SPE i.e., clean up step for the removal of matrix, when C18 is used in clean-up, being hydrophobic, C-18 retained many non-polar fatty compounds. PSA (Primary and secondary amine) exchange material having bidentate structure with strong chelating effect, used as base sorbent for d-SPE clean-up caused retention of many interfering substances like organic acids, fatty acids, sugars and other polar compounds, and it also retained some acidic sulfonyl urea herbicides (azimsulfuron, bensulfuron-methyl, ethoxysulfuron, halosulfuron methyl, pyrazosulfuron-ethyl, triasulfuron), bentazone, bispyribac sodium, bromodiolane, and imazamox thus resulting in lower recoveries (<70%). It also adsorbed polar pesticides (fipronil, lactofen, propanil, and metaflumizone) and resulted in <70% recovery (Supplementary Table S2,S3). Here PSA probably caused the formation of ionic connections with the analytes that have the negative charge, thus responsible for the loss of acidic pesticides. Hence QuEChERS citrate extraction (ME2) with 150 mg of anhydrous MgSO_4_ (MC-D) clean-up combination was further chosen to validate the method for other parameters like recovery, repeatability, etc., at 0.1 μg mL^−1^, 0.05 μg mL^−1^and 0.01 μg mL^−1^ fortification levels. Similar observations were noticed by He and Liu (2007); Lu et al. (2012) where Primary Secondary Amine (PSA) absorbed acidic pesticides like chlorpyrifos in apples and cucumbers resulting in poor recovery and false negative results.

This secondary clean-up also serves to eliminate any residual water that remains from step one and also allows extraction salts to diffuse homogenously throughout the entire sample. The end result is a more thorough, overall extraction when compared to traditional SPE protocols. Fillion et al., 1995, quantified 199 pesticides in banana, carrot, and pear samples by employing GC/MS. Small-scale charcoal-celite column clean-up is used to get rid of co-extractives. This method is tedious and time-consuming and requires a larger sample size and a lot of acetonitrile (>50 mL) per sample, and some pesticides had a large coefficient of variation due to large sample injection. In contrast, our method used QuEChERS extraction and cleanup, where only 5 mL of acetonitrile and 150 mg MgSO_4_ were used per sample (dilute sample), and the method gave >98% recovery with %RSD being <20% and Horrat values ranged from 0.2 to 0.8 for most of the pesticides.

Albero et al., 2003, quantified nine organophosphorus pesticides in fruit juices using matrix solid-phase dispersion (MSPD) of juice samples on florisil, followed by the extraction of ethyl acetate with the aid of sonication, and analysis was performed in the Gas chromatography with nitrogen-phosphorus detection. In contrast, our method has wider applicability by covering multiclass pesticides (103 pesticides) with triple quadrupole mass confirmation and sample preparation was much easier with the aid of QuEChERS.

### 3.3 Method validation

#### 3.3.1 Specificity

As per the SANTE guidelines (2021) to achieve specificity of any analyte, the peak response in reagent blank and blank control samples should be ≤30% of the fortified sample at LOQ (SANTE/11813/2021). Variations in QuEChERS extraction and clean-up combinations and different levels of dilutions were tried to ensure efficient extraction of all the fortified pesticides in the presence of undesirable interfering matrix components to ensure selective quantification. Optimization of the quantifier (Q1) and qualifier (Q2) MRM transitions, which unambiguously extracted the requisite pesticides in the presence of other pesticides and matrix interferences, allowed specificity of the pesticide for trace level identification and quantification in mango fruit drink matrix. MRM transitions for the specified pesticides under the study are given in Supplementary Table S1. The specificity of all the pesticides calculated from the peak in the reagent blank and the peak in the fortified sample at LOQ is given in Supplementary Table S4. The specificity of azoxystrobin is given in Supplementary Figure S2.

#### 3.3.2 Linearity

In the concentration range of 0.001–1 μg mL^−1^ majority of the analyte displayed linear response with correlation coefficients with r > 0.99. Diflubenzuron (0.9999), hexythiazox (0.9999), propoxur (0.9997), atrazine (0.9995), dimethoate (0.9995), pyrazosulfuron-ethyl (0.9995) had showed good linear very response with r > 0.99 whereas, fenvalerate (0.8925), cyphenothrin (0.7956), bentazone (0.7705), and Malaathio (0.6647) where observed with relatively lesser linear response with correlation coefficients (<0.80). Correlation coefficients and regression equations for all the pesticides are detailed in Supplementary Table S1 and the linearity curve for azoxystrobin is given in Supplementary Figure S3.

#### 3.3.3 Sensitivity

The sensitivity of the method was determined in terms of instrumental LOD and method LOQ after fortifying the blank matrix with the pesticide mixture at different concentration levels and subsequent processing with the developed method to achieve acceptable accuracy and precision. Method LODs and LOQs were determined to be between 0.003 and 0.3 μg mL^−1^ and 0.01–1 μg mL^−1^ respectively. Out of 103 pesticides, 81.55% (84) pesticides including anilofos, azoxystroin, butachlor, chlorpyrifos, phorate, and tebuconazolewere quantified at 0.01 μg mL^−1^ LOQ. At 0.05 μg mL^−1^ LOQ, 7.76% (8) pesticides consisting of diafenthiuron, fenvalerate, flucythrinate, isopropalin, malaathio, simazine, temephos, and tetramethrin were quantified. Whereas, 4.85% (5) of the pesticides, namely, bentazone, flubendiamide, metaflumizone, propanil, and pyriproxyfen were quantified at 0.1 μg mL^−1^. Bensufluron-methyl, bispyribac sodium, bromodiolane, ethoxysulfuron, and fipronil, on the other hand, could only be detected at 0.1 μg mL^−1^ and quizalofop-ethyl was quantified at the LOQ of 1 μg mL^−1^.

#### 3.3.4 Accuracy- recovery against the solvent standard and matrix-matched standard

Accuracy was measured in terms of recovery by fortifying different concentrations of 103 pesticidal mixtures at 0.1, 0.05, and 0.01 μg mL^−1^ (Supplementary Table S4).

At the fortified concentration of 0.1μg mL^−1^, recovery against the solvent standard (RSS) yielded 92.23% of pesticides (95 pesticides) in the acceptable range of 70%–120%, while bentazone (43.06%), bensulfuron methyl (51.40), bispyriba sodium (36.34%), ethoxysulfuron (48.05%), fipronil (36.23%), and quizalofop ethyl (55.72%) were given recovery levels less than 70%. In the case of matrix-matched standard (RMM), 91.26% of pesticides (94 pesticides) were recovered between 70% and 120%, while bentazone (56.23%), bensulfuron methyl (54.30), bispyriba sodium (31.28%), ethoxysulfuron (57.26%), fipronil (33.99%), metaflumizone (66.14%), and quizalofop ethyl (60.07%) were recovered <70%. Fenarimol had higher recoveries of >120% in both RSS and RMM. Bromodiolane was not detected in both RSS and RMM.

In the case of 0.05 μg mL^-1^ fortification, 88.34% (91) and 87.37% of the pesticides (90 pesticides) were within an acceptable range of 70%–120% for RSS and RMM, respectively. While fenarimol had >120% recovery in both RSS and RMM. Bensulfuron methyl, bispyribac sodium, ethoxysulfruon, fipronil, metaflumizone, and pyriproxyfen yielded <70% recovery in RSS. Under RMM, bensulfuron methyl, bispyribac sodium, ethoxysulfuron, fipronil, malathion, metaflumizone, and pyriproxyfen had shown a recovery of <70%. Bentazone, bromodiolane, flubendamide, propanil and quizalofop-ethyl were not at all detected in both RSS and RMM.

Out of 103 pesticides, acceptable recovery was noticed for only 77 pesticides (74.75% of the pesticides) in RSS whereas, RMM displayed an acceptable recovery for 84 pesticides (81.55% of the pesticides) respectively, when the fortification was carried at 0.01 μg mL^−1^ concentration. Herbicides like bentazone, bispyribac sodium, fipronil, isopropalin, metaflumizone, propanil, quizalofop-ethyl, simazine, and temephos and insecticides like bromodiolane, diafenthiuron, flubendiamide, malathion, and pyriproxifen, and synthetic pyrethroid group insecticides like fenvelerate, flucythrinate, and tetramethrin, were not detected in both solvent standard and matrix matched standards.

Synthetic pyrethroid insecticides (cyphenothrin, permethrin), bensulfuron methyl, chlorpyriphos-methyl, etoxysulfuron, imazamox, methomyl, and metsulfuron methyl, were shown <70% recoveries in solvent standard. Corrected recoveries were achieved using matrix-matched standards where all the above-mentioned pesticides gave acceptable recoveries of 70%–120%, except for bensulfuron methyl and ethoxysulfuron, where the recoveries were <70% in RMM.

#### 3.3.5 Precision

By calculating the HorRat ratio derived from the percentage of relative standard deviation (%RSD), the intra-laboratory repeatability for each pesticide at three fortification levels in mango fruit drink was assessed. With some exceptions, the majority of the pesticides had HorRat values between 0.2 and 0.8 (Supplementary Table S4), indicating the method’s acceptable repeatability and robustness (Horwitz et al., 1980; Horwitz and Albert, 2006). In order to extract 74 pesticides from orange juice, Rizzetti et al., 2016 developed a buffered QuEChERS extraction process employing Ultra-high-performance liquid chromatography linked to tandem mass spectroscopy (UHPLC-MS/MS). The validation findings showed the recoveries in the range of 70%–118% with an accuracy of less than 19% RSD.

#### 3.3.6 Determination of uncertainty

ISO/IEC 17025 mandates that the measurement uncertainty (U) must be established. Additionally, it must be shown that the laboratory’s own uncertainty does not go above the default value of 50% used by regulatory bodies when making enforcement decisions. The uncertainty contributors like the purity of the CRM, analytical balances, the volumetric flask used to prepare standards, micropipettes, and recovery results for all the 103 pesticides were represented in fishbone Supplementary Figure S1. The total % uncertainty of the developed method ranged from 4.72% to 23.89% where bensulfuron-methyl had the lowest (4.72%) and carfentrazone-ethyl (23.89%) had the highest % uncertainty (Supplementary Table S4). Out of 103 pesticides, 24 pesticides had % uncertainty of <10%, 64 pesticides had shown 10%–20% and 15 pesticides had uncertainty in the range of 20%–24%. As per the SANTE document (SANTE/11813/2021), when the mean bias is less than 20% and the default expanded measurement uncertainty is up to 50% it is considered acceptable at the LOQ level. In our method also all 103 pesticides had a percentage uncertainty of <24% as per SANTE recommendation, whereas 88 pesticides had a percentage uncertainty of <20%, and 15 pesticides (carbaxin, carfentrazone-ethyl, clomazone, cyphenothrin, diflubenzuron, fenamidone, flufenoxuron, fenvelerate, hexythiazox, imidacloprid, isopropalin, phosalone, profenophos, tebuconaole, and thiaclorpid) had shown <24% uncertainty of 20%–23.89% was mainly due to large variation in sample recovery, that is 10%–20% of relative standard deviation (%). This large range of uncertainty is mainly attributed to recoveries, while the rest of the parameters [uncertainty related to purity of CRM (Uc), analytical balances (Um), volumetric flask (Uf), micropipettes (Ug and Uh) (Ud and Ue), recovery (Ub), and instrument (Ua)] considered for uncertainty have not caused significant variation. The developed method is best suited for the quantification of 24 pesticides that had <10% uncertainties, and for 64 pesticides for which % uncertainty ranged from 10% to 20%, the method provides moderate performance and for the rest of the 15 pesticides, the method has a poor performance. But considering the other benefits of the developed method, special emphasis needs to be given while performing recovery studies. Similarly, Banerjee et al., 2007; Jadhav et al., 2017 reported uncertainty of up to 20% in grapes and cardamom respectively. There are no reports available so far on the determination of the method’s uncertainty in the previously established methods quoted in the manuscript (Fillion et al., 1995; Albero et al., 2003; Zhang et al., 2014; Deme and Upadhyayula, 2015; Rizzetti et al., 2016; Naz et al., 2021). Hence the present method is useful in determining the uncertainty, which is a practical strategy that encompasses trueness (bias) and reproducibility.

### 3.4 Matrix effect

In QuEChERS combined with d-SPE, the matrix effect is the major hindrance in analysing pesticide residues resulting from the matrix interference during ionization, identification, and quantification thus causing suppression or augmentation of the analytical signal. The matrix effect was prominent in the test sample, mango fruit drink, where signal enhancement was seen for most of the pesticides. Out of 103 pesticides, 77 pesticides had shown matrix enhancement where, matrix effect values were positive while 20 pesticides had shown matrix suppression of < -10% (some of the triazoles, synthetic pyrethroids, etc.). It was found that 21 pesticides had a matrix effect of <10% and 40 pesticides had matrix enhancement or suppression of 10%–20%. The matrix effect at LOQ for all the detected pesticides is given in Supplementary Table S4. In all the clean-up combinations, we could see that dilution had a considerable impact on producing acceptable recovery for numerous pesticides (Supplementary Table S4 and Figure 5), which might be due to the lowering of the matrix interference because of dilution. Banerjee et al., 2007 also found prominent matrix suppression of more than 30% for a greater number of pesticides, mostly organophosphates in grapes, and signal suppression of 20% was seen for the triazole group of pesticides. While our method had shown Matrix enhancement of >30% for 16 pesticides for some of the synthetic pyrethroids, triazoles, etc. Rajski et al. (2013) found that the matrix effect in almonds and avocado was eliminated by two and four times dilutions respectively and by the use of various sorbents such as PSA and C-18. Similar findings were reported by Ferrer et al., 2011, who found that the dilution strategy effectively eliminated the matrix effect for numerous analytes in juices like orange, leek, and tomato. However, the matrix impact was more pronounced in the presence of the matrix for particular pesticides, such as carbofuran.

### 3.5 Market sample analysis

The newly developed, single laboratory-validated Multiple reaction monitoring was employed for the estimation of pesticide residues in commercially available 10 mango drink samples in the Delhi (Indian) market. It was revealed that chlorpyrifos was detected in all the market samples, while bitertenol, tebuconazole, and tricyclazole were detected in some of the market samples of mango drinks. (Table 1). In the study, the detected pesticide residues of tebuconazole were less (<0.2 mg/kg) than the MRL values of raw mango fruit and no MRL values are available for the rest of the detected pesticides. Though CIBRC has recommended 36 pesticides including fungicides (12), insecticides (17), and plant growth regulators (7) in mango, MRL has been fixed for only 23 pesticides including a few heavy metals as per FSSAI, 2011. In the case of the mango fruit drink, neither any MRL values exist nor any systemic study is available so far in India or at the international level.

**TABLE 1:** MRL values fixed by FSSAI in raw mango and pesticide residues detected in mango fruit drink sample using the developed LC-MS/MS method.

Sl.no	Pesticide	MRL in raw mango fixed byFSSAI (mg/kg)	M_1	M_2	M_3	M_4	M_5	M_6	M_7	M_8	M_9	M_10
1	Bitertenol		-	-	-	-		-	-	0.021	-	0.010
2	Chlorpyriphos		0.023	0.025	0.021	0.022	0.023	0.028	0.028	0.022	0.022	0.020
3	Tebuconazole	**0.2**								0.020	0.011	0.011
4	Tricyclazole			0.028	0.016		0.016	0.031	0.048	0.011		0.043

M_1 to M_10 are mango drink or juice samples of various brands collected from the market.

### 3.6 GAPI (Green Analytical Procedure Index) assessment

**FIGURE 6:**
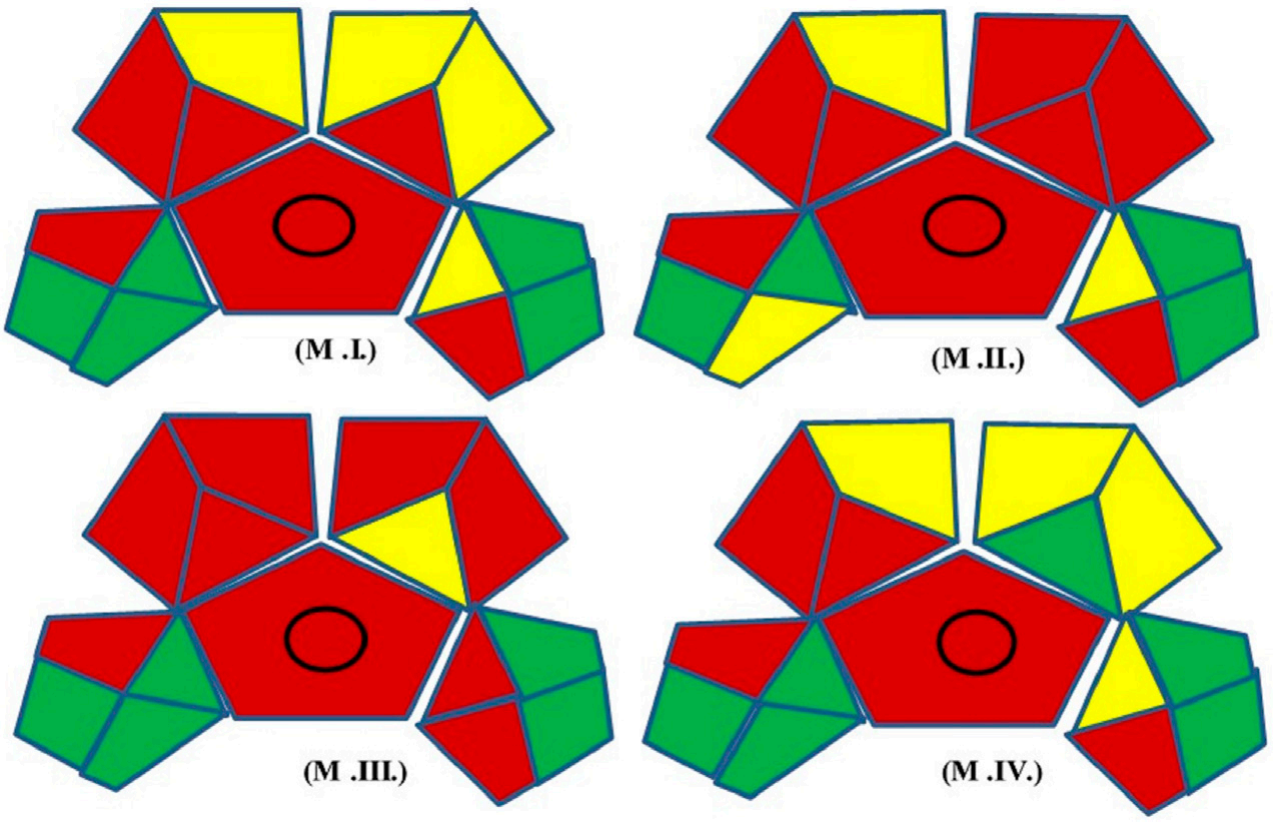
GAPI assisted comparative assessment of the green profile of the proposed method with the existed methods for the residues analysis in mango fruit drink.

Many issues have been solved by new approaches, which also increase accuracy, repeatability, throughput, and economic benefit. The ability to analyse data from samples with a reduced initial size, even at the trace level, is also essential. In the present study, the GAPI (Green Analytical Procedure Index) tool comprising pictograms of 15 various parameters is used for green assessment of the developed Multiple reaction monitoring in mango fruit drink (M.IV.). These parameters were applied for sample collection, extraction, and clean-up to final determination by the instrument and compared with three existing methods in raw mango fruit (M_I, M_II, M_III). GAPI assisted comparative assessment of the green profile of the proposed method with the existing methods for the analysis of the residues in mango fruit drink is mentioned in Figure 6 and Table 2. The developed method analysed 103 pesticides in 22 min single run, whereas M.I. analysed only one pesticide cypermethrin in 10 min run method, M. II. quantified 10 synthetic pyrethroids in 10 min run time, whereas M. III. analysed 41 pesticides in 10 min (Organochlorines, organophosphates, carbamates, and synthetic pyrethroids) (Table 3). From the analysis, it can be concluded that our developed Multiple reaction monitoring encompassing QuEChERS extraction and clean-up method (M.IV.) is safer and much more green with respect to sample preparation, solvent and reagent usage, and instrumentation than the other methods quoted in the study.

**TABLE 2:** Green Analytical Procedure Index (GAPI) Parameters and comparison between the existing method and developed method for residue analysis in Mango juice.

Index parameters	M. I	MII.		M.III.	MIV.
Sample preparation					
Collection (1)	Green	Green		Green	Green
Preservation (2)	Green	Yellow		Green	Green
Transport (3)	Green	Green		Green	Green
Storage (4)	Red	Red		Red	Red
Type of method- Sample preparation (5)	Red	Red		Red	Red
Scale of extraction (6)	Red	Red		Red	Red
Solvents/Reagents used (7)	Red	Red		Red	Red
Additional treatments (8)	Yellow	Yellow		Red	Yellow
Reagents and solvents					
Amount (9)	Red	Red		Yellow	Green
Health hazard (10)	Yellow	Red		Red	Yellow
Safety hazard (11)	Yellow	Red		Red	Yellow
Instrumentation					
Energy (12)	Yellow	Yellow		Red	Yellow
Occupational hazard (13)	Green	Green		Green	Green
Waste (14)	Green	Green		Green	Green
Waste treatment (15)	Red	Red		Red	Red
ADDITIONAL MARK- QUANTIFICATION					
Circle in the middle of GAPI: Procedure for qualification and quantification			No circle in the middle of GAPI: Procedure only for qualification		

Where, Method_1 = Naz et al., Method_2 = Zhang et al., 2014, Method_3 = Deme and Upadhyayula, 2015, Method_4 = Development, validation, and a GAPI, greenness assessment of LC-MS/MS-based method for analysis of 103 pesticides in mango fruit drink).

**TABLE 3:** List of parameters used in comparative study of the developed method with the existing methods in mango fruit drink for residue analysis.

Parameters used in analysis	M.I.	MII.	M.III.	MIV.
Method	Naz et al	Zhang et al. (2014)	Deme and Upadhyayula (2015)	Proposed method
Extraction method	SPE	QuEChERS and DLLME	d-SPE using MWCNTs	Citrate QuEChERS
Sample size (g)		15		5
Extraction buffers*/adsorbent#/solvent	Acetonitrile	Acetone, CCl_4_, ACN	MWCNTs #, Methanol, ACN, Ethyl acetate, DCM for elution. Methanol, DCM for extraction of analytes from MWCNTs	Citrate buffers*, Anhy. MgSO_4_ #, Acetonitrile
Extraction solvent volume (mL)	5 mL for conditioning of SPE, 2*2 mL for elution)	1 mL acetone, 60 µL CCl_4_ 15 mL ACN (QuEChERS)	3 mL of 40:60 v/v Methanol, DCM	5
Mobile phase solvents	Acetonitrile and water	N_2_ carrier gas	Methanol (5% toluene), water	Methanol and water
Run time (min)	10	32	10	22
Flow rate (mL/min)	1	1		0.2
Sample injection	Manual	Automated	Automated	Automated
Sample injection volume (µL)	20	1	10	2
Number of pesticides analysed	1 (Cypermethrin)	10 (Synthetic pyrethroids)	41 (Organochlorines, organophosphates, carbamates, synthetic pyrethroids)	103 (different groups of insecticides, fungicides, herbicides)
LOQ (µL/L)	<MRL (0.01 mg/kg) in mango	1–5	0.1–0.5	
Instrument	Shimadzu HPLC- PDA	Agilent GC-ECD	Thermo Scientific UPLC-APPI-HRMS	Shimadzu 8030 LC-MS/MS with ESI

* Buffers, # Adsorbent.

## 4 Conclusion

The developed method using citrate QuEChERS extraction coupled with triple quadrupole LC-MS/MS for 103 pesticides was found effective in successfully identifying and quantifying most of the pesticides fortified in mango fruit drink samples. ESI (+/−) ionization operating in MRM mode improved the selectivity and sensitivity of the pesticides. Since extraction using citrate QuEChERS buffers gave the maximum number of pesticide recoveries, this extraction method was chosen for further analysis. Dilution of mango fruit drink at different volumes prior to extraction gave good recovery for adsorbent combinations, but compared to all dilution volumes and clean-up combinations, anhy MgSO_4_ used alone in clean-up agent and 5 mL dilution gave the highest number of pesticides recovery. Matrix-matched calibration helped in compensating the matrix effect thus ensuring efficient recovery of the targeted pesticides. A single analyst can analyze roughly 20 samples in a 24-h cycle day (8 h work/day), and the instrumental method can acquire 40–42 samples per day including the run of calibrations standards for quantification. The method proved the fitness of the method as per SANTE guidelines (2021) and can be used for the intended and future purposes. The proposed method is very green in comparison with the other methods as per GAPI index parameters. Real sample analysis, i.e., mango fruit drink samples of different brands collected from the market when analyzed for residues using the developed method, gave residues for itertanol, chlorpyriphos, tricyclazole, and tebuconazole and the quantified residues of tebuconazole were less than the MRL values in raw mango fruit. However, information on MRL fixation in mango juice or mango fruit drinks is not available in both Indian and international scenarios. Hence more work needs to be done in the future to calculate the processing factor at various stages during the processing of mango into processed drinks or any other commodity, which is a crucial step in the fixation of MRL in processed mango fruit drinks and to ensure safety for human consumption.

## Data Availability

The original contributions presented in the study are included in the article/Supplementary Material, further inquiries can be directed to the corresponding author.
