# Supplementary material for: Development, validation and a GAPI greenness assessment for the determination of 103 pesticides in mango fruit drink using LC-MS/MS
Source: Front Chem. 2023 Nov 21;11:1283895. doi: 10.3389/fchem.2023.1283895 (PMC10703049; doi:10.3389/fchem.2023.1283895)
Supplement: Supplementary file 1 [file Table1.DOCX]

**Table 1. Purity of the used pesticides with their ionization mode^#^, MRL of the pesticides in raw mango, LC-MS/MS parameters, regression equations, correlation coefficients and instrumental LOD and LOQ**

| **Sl. No.** | **Pesticide**  **(Ionization mode)** | **Purity (%)** | **MRL (mg/kg)** | **RT (min)** | **Quantifier (Q1)** | **Qualifier**  **(Q2)** | **Regression Equation** | **Correlation coefficient (r)** | **(M) LOD (µg mL^-1^)** | **(M) LOQ (µg mL^-1^)** |
| --- | --- | --- | --- | --- | --- | --- | --- | --- | --- | --- |
|  |  |  |  |  |  |  |  |  |  |  |
| **Organophospates** | | | | | | | | | | |
| 1 | Anilophos(+) H | 99.6 | - | 17.7 | 368.00>125.00 | 321.90>290.10 | y=9045660x 30741.4 | 0.9928 | 0.003 | 0.01 |
| 2 | Chlorpyriphos (+) I | 99.8 | - | 16.06 | 351.85>97.05 | 305.00>153.20 | y=671515x+15567.8 | 0.9428 | 0.003 | 0.01 |
| 3 | Chlorpyriphos-methyl(+) I | 99.9 | - | 14.07 | 321.90>125.10 | 229.85>125.05 | y=146981x+4611.24 | 0.9061 | 0.003 | 0.01 |
| 4 | Diazinone(+) I | 98.5 | - | 13.19 | 305.00>169.15 | 310.90>282.90 | y=12099300x-75894.7 | 0.993 | 0.003 | 0.01 |
| 5 | Dimethoate(+) I&A | 99.5 | - | 3.79 | 229.85>199.05 | 332.05>99.10 | y=5960760x-22333.6 | 0.9995 | 0.003 | 0.01 |
| 6 | Edifenphos(+) F | 99.1 | - | 12.84 | 310.90>109.10 | 260.85>97.15 | y=6643920x-35935.2 | 0.9993 | 0.003 | 0.01 |
| 7 | Malathion(+) I | 98.7 | - | 10.62 | 332.15>128.05 | 368.00>111.05 | y=40522.0x-858.827 | 0.6647 | 0.01 | 0.05 |
| 8 | Phorate(+) I&N | 95.8 | - | 13.65 | 260.85>75.15 | 300.05>127.15 | y=482689x-1254.95 | 0.9787 | 0.003 | 0.01 |
| 9 | Phosalone(+) I&A | 99.5 | - | 13.49 | 368.00>182.05 | 372.80>304.95 | y=1896610x-12644.1 | 0.9848 | 0.003 | 0.01 |
| 10 | Phosphamidon(+) I | 97.6 | - | 5.46 | 300.05>174.15 | 299.05>163.10 | y=1963010x-11817.3 | 0.993 | 0.003 | 0.01 |
| 11 | Profenophos (+) I | 98.6 | - | 14.91 | 372.80>302.95 | 466.80>404.90 | y=1003390x-8065.71 | 0.9943 | 0.003 | 0.01 |
| 12 | Quinalphos(+) I | 99.8 | - | 12.58 | 299.05>147.15 | 299.05>163.10 | y=2937430x-3659.30 | 0.9974 | 0.003 | 0.01 |
| 13 | Temephos(+) I | 95.6 | - | 15.2 | 466.80>124.90 | 202.10>127.10 | y=1565380x-5313.37 | 0.9955 | 0.01 | 0.05 |
| **Carbamates** | | | | | | | | | | |
| 14 | Carbaryl (+) I | 97 | - | 9.2 | 202.10>145.15 | 162.90>106.10 | y=1708100x-4484.14 | 0.9925 | 0.003 | 0.01 |
| 15 | Carbofuran (+) I&A | 99.9 | - | 8.46 | 222.15>165.20 | 209.90>168.05 | y=8276220x-7437.05 | 0.9865 | 0.003 | 0.01 |
| 16 | Methomyl (+) I | 99.9 | - | 2.81 | 162.90>88.10 | 162.9>106.10 | y=560962x+6391.55 | 0.9145 | 0.003 | 0.01 |
| 17 | Propoxur (+) I | 99.8 | - | 6.29 | 209.90>111.05 | 433.15>193.00 | y=7863170x+1082.63 | 0.9997 | 0.003 | 0.01 |
| **Synthetic pyrethroids** | | | | | | | | | | |
| 18 | Alpha-Cypermethin (+) I | 98.2 | - | 14.9 | 433.15>191.00 |  | y=662301x+816.127 | 0.9993 | 0.003 | 0.01 |
| 19 | Allethrin (+) I | 96.5 | - | 14.92 | 303.00>135.10 | 467.10>224.70 | y=2300770x-11594.5 | 0.9991 | 0.003 | 0.01 |
| 20 | Bifenthrin (+) I | 99.87 | - | 15.22 | 440.00>181.12 | 393.00>123.10 | y=1458100x-60406.5 | 0.9475 | 0.003 | 0.01 |
| 21 | Cyhalothrin-lambda(+) I | 98.4 | - | 16.79 | 467.10>18.15 | 437.25>167.45 | y=446509x-3139.89 | 0.9913 | 0.003 | 0.01 |
| 22 | Cyphenothrin(+) I | 98.4 | - | 18.05 | 393.00>151.10 | 468.80>157.10 | y=5557200x+21850.6 | 0.7956 | 0.003 | 0.01 |
| 23 | Fenvalerate(+) I | 98 | - | 17.8 | 437.25>18.05 | 407.85>354.90 | y=74029.9x+229.153 | 0.8925 | 0.01 | 0.05 |
| 24 | Flucythrinate(+) I&A | 97 |  | 16.04 | 468.80>18.20 | 333.15>135.20 | y=1121180x-7921.84 | 0.9899 | 0.01 | 0.05 |
| 25 | Permethrin(+) I | 98.1 | - | 18.94 | 407.85>182.90 | 407.85>354.90 | y=3016620x-16287.9 | 0.9857 | 0.003 | 0.01 |
| 26 | Tetramethrin(+) I | 90.3 | - | 15.22 | 333.15>164.10 | 249.90>132.05 | y=335957x-1079.60 | 0.9617 | 0.01 | 0.05 |
| **Neonicotinoids** | | | | | | | | | | |
| 27 | Chlothianidine(+) I | 98.9 | - | 3.31 | 249.90>169.15 | 256.05>175.20 | y=2408110x-2321.32 | 0.9387 | 0.003 | 0.01 |
| 28 | Dinotefuron(+) I | 99.4 | - | 2.49 | 202.85>129.05 | 253.05>90.10 | y=7376020x-34128.8 | 0.9992 | 0.003 | 0.01 |
| 29 | Imidacloprid(+) I | 99.3 | 0.2 | 3.14 | 256.05>209.10 | 292.00>131.96 | y=2413350x-7164.85 | 0.987 | 0.003 | 0.01 |
| 30 | Thiacloprid(+) I | 99.7 | - | 4.03 | 253.05>126.10 | 253.05>90.10 | y=9900870x-17822.4 | 0.9961 | 0.003 | 0.01 |
| 31 | Thiamethoxam(+) I | 99.1 | 0.2 | 2.77 | 292.00>211.15 | 413.90>254.90 | y=3538720x-16060.4 | 0.9871 | 0.003 | 0.01 |
| **Phenyl pyrazole** | | | | | | | | | | |
| 32 | Ethiprole(+) I | 99.5 | - | 10.02 | 413.90>397.00 | 413.90>254.90 | y=2419250x+87442.3 | 0.9741 | 0.003 | 0.01 |
| 33 | Fipronil (-) I | 96.7 | - | 12.04 | 434.75>329.95 | 434.75>249.95 | y=265351x+12491.6 | 0.9689 | 0.3 | 1 |
| **Pyrazole** | | | | | | | | | | |
| 34 | Fenpyroximate(+) A | 98.4 | - | 17.12 | 421.90>366.13 | 248.00>93.05 | y=23108600-112012 | 0.9995 | 0.003 | 0.01 |
| **Phenyl urea** | | | | | | | | | | |
| 35 | Forchlofenuron(+) PGR | 99.9 | - | 8.52 | 248.00>129.05 | 248.00>93.05 | y=12705800x-71072.1 | 0.9956 | 0.003 | 0.01 |
| 36 | Pencycuron(+) F | 99.7 | - | 13.76 | 329.05>125.05 | 238.95>16.55 | y=14537200x-47534.7 | 0.9988 | 0.003 | 0.01 |
| **Thiadiazines** | | | | | | | | | | |
| 37 | Bentazone (-) H | 99.9 | - | 3.1 | 238.95>131.90 | 238.95>16.55 | y=97402.8x+4329.91 | 0.7705 | 0.03 | 0.1 |
| 38 | Buprofezin(+) I | 99.1 | 0.1 | 14.92 | 306.00>57.15 | 322.00>185.10 | y=16233100x-53575.1 | 0.9951 | 0.003 | 0.01 |
| **Pyridine** | | | | | | | | | | |
| 39 | Pyriproxyfen(+) I | 99 | - | 15.77 | 322.00>91.20 | 307.05>161.20 | y=423348x+1638.04 | 0.9739 | 0.03 | 0.1 |
| **Quinazoline** | | | | | | | | | | |
| 40 | Fenazaquin(+) I&A | 99.9 | - | 18.38 | 307.05>57.15 | 527.95>150.05 | y=26788900x-109907 | 0.9989 | 0.003 | 0.01 |
| **Oxadiazine** | | | | | | | | | | |
| 41 | Indoxacarb(+) I | 93.6 | - | 13.98 | 527.95>56.05 | 483.80>286.00 | y=1003490x-4669.42 | 0.9994 | 0.003 | 0.01 |
| **Ryanoid compounds** | | | | | | | | | | |
| 42 | Chlorantraliprole(+) I | 96.9 | - | 9 | 483.80>453.00 | 483.80>286.00 | y=1614750x-6667.12 | 0.9971 | 0.003 | 0.01 |
| 43 | Flubendiamide(-) I | 99.9 | - | 12.32 | 681.05>254.10 | 310.75>140.90 | y=25543.6x+205.171 | 0.9841 | 0.03 | 0.1 |
| **Benzophenyl urea** | | | | | | | | | | |
| 44 | Diflubenzuron(+) I | 98.1 | - | 12 | 310.75>157.95 | 310.75>140.90 | y=8820510x-64453.3 | 0.9999 | 0.003 | 0.01 |
| 45 | Flufenoxuron (+) I | 98.4 | - | 16.19 | 488.90>158.05 | 384.95>169.25 | y=7193540x-59843.2 | 0.9991 | 0.003 | 0.01 |
| **Thiourea** | | | | | | | | | | |
| 46 | Diafenthiuron(+) I&A | 99.9 | - | 16.96 | 384.95>329.25 | 384.95>169.25 | y=387529x-842.502 | 0.9528 | 0.01 | 0.05 |
| 47 | Methabenzthiazuron (+) H | 98 | - | 6.39 | 222.00>165.10 | 505.10>117.05 | y=15540600x-26132.7 | 0.9978 | 0.003 | 0.01 |
| **Semicarbazone** | | | | | | | | | | |
| 48 | Metaflumizone(+) I | 99.3 | - | 14.97 | 505.10>178.00 | 353.10>168.10 | y=857972x-5775.14 | 0.9859 | 0.03 | 0.1 |
| **Carboxamide** | | | | | | | | | | |
| 49 | Hexythiazox(+) I | 99.9 | - | 16.02 | 353.10>228.10 | 353.10>168.10 | y=4335950x-29814.0 | 0.9999 | 0.003 | 0.01 |
| **Coumarin** | | | | | | | | | | |
| 50 | Bromodiolone(+) R | 96.1 | - | 17.71 | 526.65>260.80 | 404.00>344.20 | y=84300.5x-6689.01 | 0.9899 | 0.3 | 1 |
| **Strobilurins** | | | | | | | | | | |
| 51 | Azoxystrobin(+) F | 99.4 | 0.7 | 13.05 | 404.00>372.20 | 388.10>163.15 | y=11163900x-32226.6 | 0.9989 | 0.003 | 0.01 |
| 52 | Kresoxim methyl(+) F | 96 | - | 12.61 | 314.15>267.15 | 314.15>222.15 | y=907479x-1445.66 | 0.9908 | 0.003 | 0.01 |
| 53 | Pyraclostrobin(+) F | 99.9 | - | 13.29 | 388.10>194.15 | 338.10>269.10 | y=4947880x-11162.2 | 0.9996 | 0.003 | 0.01 |
| **Triazoles** | | | | | | | | | | |
| 54 | Bitertenol(+) F | 99.4 | - | 19.21 | 338.10>99.10 | 315.95>165.10 | y=4264670x-17969.0 | 0.9984 | 0.003 | 0.01 |
| 55 | Difenoconazole (+) F | 95.9 | - | 13.92 | 407.95>251.00 | 313.95>159.05 | y=8403410x-38345.4 | 0.9996 | 0.003 | 0.01 |
| 56 | Flusilazole (+) F | 99.8 | - | 12.12 | 315.95>247.10 | 289.05>125.10 | y=7708110x-55126.0 | 0.9986 | 0.003 | 0.01 |
| 57 | Hexaconazole(+) F | 99.3 | 0.02 | 13.33 | 313.95>70.15 | 284.10>70.10 | y=7172170x-42238.4 | 0.9987 | 0.003 | 0.01 |
| 48 | Myclobutanil(+) F | 99.4 | - | 10.85 | 289.05>70.10 | 341.95>159.05 | y=5686080x-32903.6 | 0.997 | 0.003 | 0.01 |
| 59 | Penconazole(+) F | 97.7 | - | 12.72 | 285.10>70.10 | 308.00>125.10 | y=3303280x-18242.3 | 0.9887 | 0.003 | 0.01 |
| 60 | Propiconazole(+) F | 98.4 | - | 13.14 | 341.95>69.10 | 189.95>163.05 | y=4750940x-26129.5 | 0.9996 | 0.003 | 0.01 |
| 61 | Tebuconazole(+) F | 98.7 | 0.2 | 12.82 | 308.00>70.10 | 294.00>225.10 | y=12089500x-67339.8 | 0.992 | 0.003 | 0.01 |
| 62 | Tricyclazole(+) F | 99.4 | - | 4.72 | 189.95>136.05 | 189.95>163.05 | y=4173970x-31.8477 | 0.9851 | 0.003 | 0.01 |
| 63 | Triadimefon(+) F | 99.5 | 0.03 | 10.89 | 294.00>69.10 | 192.10>132.15 | y=7140760x-48235.8 | 0.9968 | 0.003 | 0.01 |
| **Benzimidazole** | | | | | | | | | | |
| 64 | Carbendazim(+) F | 98.2 | 2 | 5.88 | 192.10>160.15 | 236.05>86.95 | y=12556400x+12333 | 0.9903 | 0.003 | 0.01 |
| **Oxathiin carboxamide** | | | | | | | | | | |
| 65 | Carboxin(+) F | 99.9 | - | 9.24 | 236.05>143.05 | 236.25>86.95 | y=8682090x-37544.6 | 0.9971 | 0.003 | 0.01 |
| 66 | Oxycarboxin(+) F | 99.9 | - | 4.12 | 267.95>175.05 | 335.80>195.80 | y=13474300x-48291.6 | 0.9996 | 0.003 | 0.01 |
| **Carbaxamide** | | | | | | | | | | |
| 67 | Carpropamid(+) F | 99.9 | - | 12.9 | 335.80>138.90 | 291.00>189.05 | y=4185380x-28938.6 | 0.9964 | 0.003 | 0.01 |
| **Dithiolane** | | | | | | | | | | |
| 68 | Isoprothiolane(+) F | 99.1 | - | 10.64 | 291.00>231.05 | 199.10>111.05 | y=9527470x-33331.7 | 0.9951 | 0.003 | 0.01 |
| **Acetamide** | | | | | | | | | | |
| 69 | Cymoxanil(+) F | 99.4 | - | 4.26 | 199.10>128.05 | 388.10>165.15 | y=1198830x+9972.73 | 0.9808 | 0.003 | 0.01 |
| **Morpholine** | | | | | | | | | | |
| 70 | Dimethomorph(+) F | 99 | - | 10.52 | 388.10>301.05 | 312.05>236.20 | y=7303000x-51139.8 | 0.9985 | 0.003 | 0.01 |
| **Pyrimidine** | | | | | | | | | | |
| 71 | Fenamidone(+) F | 99.9 | - | 10.01 | 312.05>92.10 | 331.10>81.10 | y=9973700x-66857.5 | 0.9958 | 0.003 | 0.01 |
| **Imidazolones** | | | | | | | | | | |
| 72 | Fenarimol(+) F | 99.9 | - | 11.48 | 331.90>81.10 | 321.15>203.10 | y=397888x+14388.8 | 0.9704 | 0.03 | 0.01 |
| **Amide carbamate** | | | | | | | | | | |
| 73 | Iprovalicarb(+) F | 98.7 | - | 11.39 | 321.15>119.10 | 280.05>192.15 | y=13508400x-62452.8 | 0.9972 | 0.003 | 0.01 |
| **Acyl alanine** | | | | | | | | | | |
| 74 | Metalaxyl (+) F | 99.6 | - | 8.52 | 280.05>220.15 | 430.65>412.95 | y=11349200x-55289.8 | 0.997 | 0.003 | 0.01 |
| **Benzoic acid** | | | | | | | | | | |
| 75 | Bispyribac sodium(+) H | 99.4 | - | 7.58 | 430.65>274.90 | 430.60>367.85 | y=9461080x-73707.1 | 0.9982 | 0.3 | 1 |
| **Aryl triazone** | | | | | | | | | | |
| 76 | Carfentrazone ethyl(+) H | 96.7 | - | 17.22 | 430.60>413.90 | 240.00>89.10 | y=1194220x+35120.4 | 0.9994 | 0.003 | 0.01 |
| **Isoxazolidonone** | | | | | | | | | | |
| 77 | Clomazone(+) H | 98.3 | - | 9.42 | 240.00>125.05 | 364.00>194.15 | y=9197700x-66492.2 | 0.9909 | 0.003 | 0.01 |
| **Oxiacetamide** | | | | | | | | | | |
| 78 | Flufenacet(+) H | 99.7 | - | 11.56 | 364.00>152.10 | 364.00>194.15 | y=9424160x-50337.1 | 0.9983 | 0.003 | 0.01 |
| 79 | Imazamox(+) H | 99.9 | - | 2.33 | 305.90>261.00 | 478.75>222.75 | y=1973660x-7627.33 | 0.9686 | 0.003 | 0.01 |
| **Nitrophenyl ether** | | | | | | | | | | |
| 80 | Lactofen(+) H& F | 96.2 | - | 15.04 | 478.75>343.85 | 350.20>238.00 | y=11841700x-94169.2 | 0.9993 | 0.003 | 0.01 |
| **Phenoxy acid ester** | | | | | | | | | | |
| 81 | Clodinafop-propargyl(+) H | 99.2 | - | 12.44 | 350.20>266.00 | 358.10>120.00 | y=146977x+395.537 | 0.9937 | 0.003 | 0.01 |
| 82 | Cyhalofop butyl(+) H | 99.1 | - | 14.14 | 374.75>255.90 | 362.20>76.90 | y=1359980x-33780.6 | 0.9968 | 0.003 | 0.01 |
| 83 | Diclofop-methyl(+) H | 99.1 | - | 15.17 | 358.10>281.00 | 384.10>328.00 | y=490982x+9970.85 | 0.9986 | 0.003 | 0.01 |
| 84 | Fenoxaprop-p-ethyl (+) H | 99.1 | - | 14.8 | 362.20>288.00 | 376.10>315.95 | y=129042x+712.878 | 0.9878 | 0.003 | 0.01 |
| 85 | Fluazifop-p-butyl(+) H | 96.1 | - | 14.95 | 384.10>282.20 | 373.10>271.10 | y=3314260x-14620.7 | 0.9968 | 0.003 | 0.01 |
| 86 | Haloxyfop methyl(+) H | 99.9 | - | 14.07 | 376.10>90.85 | 376.10>315.95 | y=411301x+796.018 | 0.997 | 0.003 | 0.01 |
| 87 | Quizalofop ethyl(+) H | 96.3 | - | 14.94 | 373.10>299.40 | 424.70>82.90 | y=15266.8x+1063.42 | 0.9481 | 0.3 | 1 |
| **Sulfonyl urea** | | | | | | | | | | |
| 88 | Azimsulfuron (+) H | 100 | - | 3.44 | 424.70>181.95 | 399.10>218.20 | y=8559190x-71205.6 | 0.9814 | 0.003 | 0.01 |
| 89 | Bensulfuron-methyl (+) H | 99 | - | 8.74 | 410.80>148.90 | 435.00>83.10 | y=15852600x-10788.3 | 0.9998 | 0.3 | 1 |
| 90 | Ethoxysulfuron(+) H | 97.3 | - | 6.09 | 399.10>261.10 | 382.00>141.00 | y=2365980x-16160.9 | 0.991 | 0.3 | 1 |
| 91 | Halosulfuron-methyl(+) H | 99.9 | - | 4.38 | 435.00>182.10 | 415.10>83.00 | y=4729960x-25849.8 | 0.9965 | 0.003 | 0.01 |
| 92 | Metsulfuron-methyl(+) H | 98.7 | - | 2.67 | 382.00>167.10 | 401.70>141.95 | y=9336590x-75760.9 | 0.99 | 0.003 | 0.01 |
| 93 | Pyrazosulfuron-ethyl (+) H | 99.6 | - | 4.95 | 415.10>182.10 | 415.10>83.00 | y=6637720x-42284.3 | 0.9995 | 0.003 | 0.01 |
| 94 | Triasulfuron(+) H | 97.1 | - | 3.79 | 401.70>166.95 | 312.10>87.00 | y=5210110x-19835.8 | 0.9894 | 0.003 | 0.01 |
| **Anilides** | | | | | | | | | | |
| 95 | Butachlor(+) H | 99 | - | 14.93 | 312.10>238.00 | 212.00>169.90 | y=1447830x+2096.09 | 0.9906 | 0.003 | 0.01 |
| 96 | Pretilachlor(+) H | 98.2 | - | 14.67 | 311.90>251.95 | 216.00>126.80 | y=23495300x-97903.1 | 0.9988 | 0.003 | 0.01 |
| 97 | Propachlor(+) H | 99.8 | - | 8.47 | 212.00>169.90 | 212.00>169.90 | y=4566160x-26348.6 | 0.9913 | 0.003 | 0.01 |
| 98 | Propanil(-) H | 99.6 | - | 9.99 | 216.00>159.80 | 216.00>126.80 | y=32607.7x+65179.4 | 0.9841 | 0.03 | 0.1 |
| **Dinitroalines** | | | | | | | | | | |
| 99 | Isopropalin(+) H | 97.2 | - | 16.9 | 310.20>226.00 |  | y=240158x+3630.66 | 0.9967 | 0.01 | 0.05 |
| 100 | Pendimethalin(+) H | 98.8 | - | 16.21 | 281.90>211.80 | 216.00>132.00 | y=4119120x+20347.3 | 0.9881 | 0.003 | 0.01 |
| **Triazines** | | | | | | | | | | |
| 101 | Atrazine (+) H | 98.1 | - | 10.97 | 216.00>174.20 | 201.90>103.85 | y=3831650x-17872.8 | 0.9995 | 0.003 | 0.01 |
| 102 | Propazine(+) H | 99.3 | - | 9.893 | 230.00>146.10 | 368.00>199.00 | y=6136370x-51301.7 | 0.9938 | 0.003 | 0.01 |
| 103 | Simazine(+) H | 99.7 | - | 6.52 | 201.90>124.00 | 201.90>103.85 | y=2566930x-17886.5 | 0.9952 | 0.01 | 0.05 |
|  |  |  |  |  |  |  |  |  |  |  |

I = Insecticide, F =Fungicide, H =Herbicide, A=Acaricide, N=Nematicide, PGR=Plant growth regulator, #Ionization mode positive (+) or negative (-) are given in parenthesis, MRL =Maximum Residue Limit, RT = Retention time, CE = Collision Energy, (M). LOD=Method Limit of Detection, (M). LOQ= Method Limit of Quantification.

**Table S2. Recovery percentage of fortified pesticides at 1 µg g^-1^ in mango fruit drink by various QuEChERS extraction (original, citrate and acetate buffered) and clean-up combinations**

|  | **ME1** | | | | **ME2** | | | | **ME3** | | | |
| --- | --- | --- | --- | --- | --- | --- | --- | --- | --- | --- | --- | --- |
| **Pesticides** | **MC-A** | **MC-B** | **MC-C** | **MC-D** | **MC-A** | **MC-B** | **MC-C** | **MC-D** | **MC-A** | **MC-B** | **MC-C** | **MC-D** |
| Alpha-Cypermethin | 58.53 | 117.28 | 85.49 | 108.72 | 70.73 | 75.60 | 80.99 | 72.14 | 110.22 | 129.75 | 109.09 | 108.88 |
| Allethrin | 76.15 | 89.10 | 106.42 | 93.22 | 74.86 | 77.86 | 75.82 | 71.70 | 73.92 | 76.01 | 99.57 | 97.20 |
| Anilophos | 86.15 | 109.84 | 101.67 | 79.11 | 85.03 | 79.89 | 80.50 | 81.01 | 85.31 | 89.12 | 93.00 | 83.80 |
| Atrazine | 80.21 | 107.74 | 107.65 | 98.60 | 101.39 | 98.15 | 95.01 | 92.56 | 84.46 | 83.38 | 106.13 | 92.32 |
| Azimsulfuron | 15.60 | 112.42 | 23.55 | 101.28 | 23.39 | 25.71 | 9.26 | 107.01 | 11.80 | 13.04 | 7.09 | 111.83 |
| Azoxystrobin | 67.63 | 100.19 | 102.05 | 96.34 | 84.83 | 82.68 | 77.36 | 75.01 | 74.43 | 78.15 | 85.58 | 78.93 |
| Bensulfuron-methyl | 26.90 | 91.27 | 21.82 | 61.17 | 23.16 | 22.94 | 8.93 | 72.68 | 58.98 | 52.89 | 21.22 | 90.54 |
| Bentazone | 34.95 | 1.24 | 36.51 | 46.45 | 24.44 | 24.09 | 9.59 | 7.22 | 10.20 | 13.42 | 6.98 | 58.38 |
| Bifenthrin | 62.29 | 112.26 | 89.61 | 92.65 | 71.42 | 76.77 | 80.62 | 73.13 | 69.41 | 79.35 | 91.33 | 92.19 |
| Bispyribac sodium | 0.71 | 96.94 | 1.00 | 76.45 | 0.67 | 0.66 | 0.14 | 2.63 | 0.27 | 0.42 | 0.39 | 50.80 |
| Bitertenol | 86.04 | 94.80 | 105.25 | 91.79 | 138.48 | 86.93 | 85.49 | 83.33 | 89.68 | 88.35 | 87.54 | 89.38 |
| Bromodiolone | - | 104.76 | - | 75.55 | - | - | - | - | - | - | - | 112.67 |
| Buprofezin | 63.85 | 96.88 | 98.16 | 91.22 | 84.42 | 84.94 | 82.47 | 78.10 | 75.51 | 76.31 | 93.65 | 87.82 |
| Butachlor | 67.82 | 106.40 | 93.20 | 86.58 | 82.98 | 80.62 | 82.39 | 77.91 | 66.95 | 70.10 | 84.77 | 80.68 |
| Carbaryl | 87.14 | 107.46 | 98.01 | 72.01 | 76.01 | 72.66 | 68.96 | 71.58 | 75.79 | 75.87 | 79.85 | 71.37 |
| Carbendazim | 73.46 | 117.59 | 95.53 | 85.35 | 103.87 | 102.03 | 94.24 | 91.65 | 331.64 | 311.43 | 276.33 | 293.09 |
| Carbofuran | 84.91 | 116.84 | 103.33 | 71.68 | 82.73 | 78.29 | 75.90 | 74.29 | 117.08 | 111.70 | 109.36 | 103.87 |
| Carboxin | 89.96 | 99.66 | 103.21 | 61.56 | 73.60 | 72.96 | 70.75 | 70.68 | 86.08 | 85.19 | 83.73 | 74.48 |
| Carfentrazone ethyl | 80.98 | 93.69 | 114.78 | 107.98 | 85.54 | 84.22 | 80.69 | 82.37 | 95.38 | 99.33 | 97.24 | 92.35 |
| Carpropamid | 103.88 | 129.53 | 124.22 | 87.91 | 92.64 | 90.66 | 85.18 | 79.31 | 96.73 | 96.46 | 111.39 | 95.43 |
| Chlorantraliprole | 90.87 | 121.05 | 108.14 | 73.74 | 86.83 | 82.76 | 78.65 | 84.66 | 85.09 | 85.84 | 92.10 | 85.16 |
| Chlorpyriphos | 71.36 | 96.58 | 89.61 | 80.86 | 83.52 | 89.83 | 91.60 | 87.94 | 74.18 | 79.24 | 92.63 | 86.44 |
| Chlorpyriphos-methyl | 76.03 | 107.95 | 94.00 | 72.77 | 74.98 | 70.98 | 69.70 | 70.37 | 80.15 | 83.87 | 85.07 | 78.59 |
| Chlothianidine | 98.44 | 99.69 | 124.92 | 99.20 | 115.14 | 113.48 | 112.79 | 109.72 | 98.59 | 93.81 | 113.01 | 91.83 |
| Clodinafop-propargyl | 101.86 | 114.69 | 113.69 | 77.06 | 81.72 | 79.44 | 75.35 | 73.23 | 86.44 | 88.61 | 88.16 | 83.29 |
| Clomazone | 68.46 | 101.57 | 98.79 | 93.05 | 90.74 | 90.01 | 88.68 | 86.32 | 79.13 | 79.80 | 91.04 | 90.58 |
| Cyhalofop butyl | 73.64 | 99.63 | 104.13 | 112.33 | 83.56 | 77.25 | 79.74 | 73.63 | 77.16 | 78.89 | 98.45 | 93.43 |
| Cyhalothrin-lambda | 78.21 | 97.95 | 89.63 | 74.30 | 60.80 | 65.98 | 67.79 | 86.22 | 66.52 | 72.26 | 86.57 | 88.03 |
| Cymoxanil | 93.97 | 95.01 | 115.02 | 83.34 | 84.62 | 82.56 | 79.04 | 74.66 | 92.58 | 91.67 | 93.26 | 83.48 |
| Cyphenothrin | 75.34 | 99.91 | 94.27 | 77.72 | 62.19 | 67.99 | 66.78 | 74.26 | 68.77 | 79.71 | 102.97 | 104.03 |
| Diafenthiuron | 73.76 | 119.09 | 94.54 | 78.73 | 81.05 | 80.58 | 75.12 | 75.95 | 72.28 | 74.84 | 94.56 | 85.09 |
| Diazinone | 72.16 | 104.97 | 97.66 | 84.88 | 89.32 | 86.39 | 84.67 | 81.65 | 75.71 | 77.89 | 89.83 | 80.88 |
| Diclofop-Methyl | 64.34 | 119.60 | 98.31 | 110.17 | 86.08 | 86.80 | 88.85 | 84.63 | 81.27 | 87.46 | 94.54 | 93.09 |
| Difenoconazole | 64.91 | 110.43 | 101.49 | 119.74 | 96.01 | 91.67 | 94.04 | 92.18 | 84.57 | 85.73 | 104.13 | 100.88 |
| Diflubenzuron | 91.67 | 129.41 | 111.38 | 85.40 | 78.74 | 75.51 | 75.02 | 74.30 | 82.38 | 81.73 | 92.42 | 85.77 |
| Dimethoate | 74.91 | 17.82 | 107.69 | 92.73 | 83.94 | 83.75 | 77.34 | 76.84 | 85.26 | 84.57 | 94.49 | 88.75 |
| Dimethomorph | 99.85 | 122.94 | 110.17 | 72.61 | 86.17 | 83.78 | 84.21 | 80.45 | 86.19 | 87.01 | 86.04 | 83.97 |
| Dinotefuron | 110.13 | 135.99 | 117.07 | 66.59 | 71.50 | 67.75 | 61.04 | 70.59 | 50.08 | 49.68 | 48.70 | 43.51 |
| Edifenphos | 75.94 | 86.56 | 107.32 | 100.69 | 87.27 | 83.32 | 77.79 | 79.24 | 64.09 | 66.11 | 73.83 | 69.92 |
| Ethiprole | 96.05 | 107.11 | 125.91 | 115.02 | 95.12 | 92.26 | 90.01 | 91.68 | 48.45 | 50.62 | 55.03 | 49.54 |
| Ethoxysulfuron | 10.36 | 101.92 | 12.43 | 51.54 | 9.96 | 11.11 | 4.35 | 49.57 | 6.42 | 7.29 | 3.45 | 100.91 |
| Fenamidone | 97.50 | 102.11 | 116.59 | 90.73 | 98.01 | 103.26 | 100.53 | 96.36 | 97.58 | 95.75 | 109.48 | 97.42 |
| Fenarimol | 113.16 | 101.45 | 117.98 | 91.98 | 96.56 | 99.33 | 92.29 | 95.00 | 103.37 | 97.86 | 103.65 | 97.25 |
| Fenazaquin | 55.28 | 104.49 | 90.22 | 98.25 | 81.23 | 81.51 | 77.50 | 78.03 | 67.75 | 68.33 | 87.62 | 86.51 |
| Fenoxaprop-p-ethyl | 90.69 | 108.44 | 96.58 | 66.89 | 74.21 | 77.32 | 74.82 | 74.94 | 72.87 | 75.78 | 81.09 | 72.71 |
| Fenpyroximate | 69.36 | 87.06 | 101.06 | 88.17 | 83.97 | 79.94 | 76.35 | 75.56 | 82.24 | 73.69 | 90.50 | 80.59 |
| Fenvalerate | 82.76 | 110.87 | 91.49 | 77.85 | 58.12 | 66.62 | 64.28 | 74.45 | 61.33 | 73.71 | 83.86 | 85.06 |
| Fipronil | 84.08 | 120.02 | 98.57 | 64.69 | 59.28 | 54.57 | 54.08 | 47.99 | 76.29 | 73.14 | 72.44 | 67.91 |
| Fluazifop-p-butyl | 83.61 | 51.25 | 97.03 | 70.11 | 77.23 | 74.32 | 75.28 | 70.40 | 72.88 | 76.40 | 90.41 | 76.10 |
| Flubendiamide | 113.46 | 99.78 | 111.94 | 49.24 | 57.47 | 51.94 | 47.25 | 59.94 | 78.40 | 78.58 | 81.69 | 60.98 |
| Flucythrinate | 66.70 | 121.62 | 101.99 | 103.91 | 52.92 | 50.97 | 47.75 | 61.35 | 71.24 | 76.28 | 85.45 | 91.39 |
| Flufenacet | 79.32 | 95.77 | 102.97 | 90.00 | 93.49 | 91.12 | 93.02 | 87.83 | 78.98 | 82.20 | 94.57 | 87.91 |
| Flufenoxuron | 61.80 | 137.49 | 83.63 | 94.81 | 71.39 | 75.75 | 77.68 | 74.03 | 62.87 | 72.70 | 91.60 | 105.18 |
| Flusilazole | 78.15 | 113.82 | 110.45 | 106.16 | 93.83 | 90.69 | 88.56 | 84.97 | 79.23 | 80.23 | 95.18 | 89.75 |
| Forchlofenuron | 97.94 | 104.68 | 111.75 | 95.14 | 107.04 | 109.44 | 99.04 | 119.09 | 90.47 | 91.75 | 90.84 | 108.58 |
| Halosulfuron-methyl | 31.34 | 79.87 | 33.53 | 72.16 | 29.62 | 33.32 | 14.12 | 95.11 | 8.39 | 10.34 | 7.50 | 84.34 |
| Haloxyfop methyl | 67.17 | 106.75 | 99.56 | 113.84 | 82.25 | 82.47 | 82.04 | 79.24 | 79.21 | 83.80 | 93.68 | 86.95 |
| Hexaconazole | 87.94 | 110.53 | 117.02 | 118.01 | 110.27 | 107.15 | 106.13 | 104.59 | 94.20 | 96.51 | 111.34 | 109.40 |
| Hexythiazox | 72.32 | 115.81 | 90.05 | 78.16 | 76.50 | 78.68 | 83.43 | 76.76 | 69.30 | 74.48 | 89.46 | 90.94 |
| Imazamox | 1.67 | 120.08 | 1.28 | 59.68 | 0.91 | 1.40 | - | 83.76 | - | 0.57 | - | 32.94 |
| Imidacloprid | 127.96 | 101.85 | 127.06 | 78.53 | 111.17 | 113.94 | 108.13 | 103.62 | 99.06 | 97.96 | 108.27 | 84.75 |
| Indoxacarb | 79.82 | 103.92 | 110.24 | 90.38 | 72.38 | 70.74 | 65.03 | 75.06 | 84.08 | 84.86 | 94.27 | 93.71 |
| Iprovalicarb | 82.14 | 106.58 | 97.38 | 71.59 | 79.59 | 83.71 | 79.91 | 80.37 | 72.29 | 72.23 | 78.88 | 70.82 |
| Isopropalin | 50.71 | 115.54 | 82.01 | 97.15 | 70.08 | 76.20 | 77.37 | 71.16 | 60.49 | 70.31 | 78.86 | 79.39 |
| Isoprothiolane | 81.45 | 26.79 | 103.21 | 86.10 | 88.85 | 91.54 | 88.40 | 88.66 | 79.90 | 81.22 | 94.62 | 82.83 |
| Kresoxim methyl | 83.65 | 120.58 | 106.96 | 86.28 | 88.37 | 83.12 | 82.79 | 81.09 | 80.40 | 80.79 | 86.62 | 80.66 |
| Lactofen | 82.75 | 105.89 | 111.73 | 109.02 | 71.84 | 67.53 | 64.86 | 76.85 | 71.89 | 78.84 | 92.07 | 93.11 |
| Malathion | 110.16 | 109.02 | 104.48 | 61.38 | 72.94 | 76.67 | 73.08 | 71.34 | 84.59 | 81.71 | 82.98 | 72.92 |
| Metaflumizone | 68.87 | 104.74 | 105.97 | 104.37 | 74.02 | 62.22 | 58.14 | 70.42 | 71.27 | 74.52 | 75.72 | 81.35 |
| Metalaxyl | 74.46 | 98.84 | 106.52 | 92.91 | 75.75 | 71.92 | 69.04 | 70.69 | 77.32 | 75.57 | 73.91 | 71.81 |
| Methabenzthiazuron | 75.37 | 98.67 | 105.79 | 97.45 | 96.58 | 98.27 | 97.66 | 95.47 | 77.44 | 79.32 | 88.79 | 83.28 |
| Methomyl | 84.34 | 101.99 | 110.37 | 78.12 | 79.57 | 77.62 | 73.81 | 71.77 | 31.58 | 33.30 | 37.70 | 34.57 |
| Metsulfuron-methyl | 14.97 | 103.44 | 18.50 | 94.62 | 18.41 | 20.43 | 7.37 | 103.26 | 13.91 | 14.18 | 6.60 | 119.42 |
| Myclobutanil | 108.55 | 96.46 | 111.80 | 73.10 | 80.71 | 83.13 | 82.00 | 83.53 | 87.52 | 88.54 | 85.37 | 79.40 |
| Oxycarboxin | 82.78 | 104.00 | 80.78 | 97.17 | 85.68 | 77.28 | 52.45 | 84.96 | 88.09 | 85.47 | 69.63 | 86.31 |
| Penconazole | 78.97 | 104.27 | 112.09 | 116.40 | 101.11 | 93.24 | 93.96 | 93.86 | 92.93 | 96.45 | 103.68 | 99.48 |
| Pencycuron | 76.36 | 108.66 | 102.49 | 90.83 | 88.85 | 91.70 | 85.43 | 86.53 | 74.78 | 75.82 | 92.10 | 86.01 |
| Pendimethalin | 74.18 | 110.38 | 91.96 | 77.71 | 72.79 | 73.24 | 76.05 | 70.31 | 67.19 | 76.42 | 94.92 | 95.64 |
| Permethrin | 70.89 | 114.54 | 92.93 | 94.26 | 67.92 | 76.58 | 78.92 | 70.64 | 63.68 | 73.52 | 95.39 | 96.82 |
| Phorate | 71.54 | 113.33 | 98.58 | 79.67 | 70.62 | 72.26 | 65.65 | 78.22 | 52.81 | 55.99 | 63.48 | 56.93 |
| Phosalone | 77.53 | 105.44 | 105.02 | 97.44 | 79.95 | 76.83 | 74.72 | 72.23 | 79.18 | 84.98 | 88.68 | 85.26 |
| Phosphamidon | 83.90 | 38.82 | 95.64 | 57.80 | 73.00 | 71.90 | 64.91 | 71.57 | 67.78 | 67.59 | 74.24 | 60.70 |
| Pretilachlor | 78.45 | 86.27 | 100.70 | 76.44 | 73.73 | 71.80 | 69.06 | 78.96 | 70.25 | 68.39 | 84.15 | 73.71 |
| Profenophos | 73.93 | 107.68 | 102.66 | 112.58 | 99.58 | 96.45 | 93.54 | 96.01 | 74.68 | 85.09 | 117.43 | 110.02 |
| Propachlor | 75.96 | 116.42 | 110.42 | 98.44 | 88.99 | 88.45 | 84.51 | 80.70 | 67.10 | 67.63 | 82.50 | 73.02 |
| Propanil | 86.88 | 119.46 | 107.90 | 60.25 | 67.19 | 56.02 | 52.99 | 46.23 | 72.60 | 68.84 | 71.66 | 67.94 |
| Propazine | 78.03 | 126.36 | 108.06 | 100.01 | 89.54 | 85.67 | 82.27 | 79.05 | 80.56 | 79.95 | 90.46 | 82.08 |
| Propiconazole | 81.57 | 111.30 | 109.42 | 112.92 | 103.60 | 100.08 | 98.81 | 97.54 | 91.85 | 90.60 | 107.42 | 102.33 |
| Propoxur | 92.24 | 152.33 | 106.86 | 65.52 | 76.90 | 74.73 | 72.62 | 71.13 | 73.14 | 72.66 | 78.10 | 70.95 |
| Pyraclostrobin | 80.69 | 107.72 | 99.75 | 76.21 | 82.92 | 80.69 | 79.51 | 79.16 | 76.12 | 75.00 | 83.53 | 75.89 |
| Pyrazosulfuron-ethyl | 22.09 | 110.08 | 27.66 | 66.71 | 22.98 | 24.98 | 8.96 | 79.47 | 11.77 | 13.15 | 6.43 | 83.11 |
| Pyriproxyfen | 58.30 | 32.00 | 89.32 | 95.57 | 93.84 | 92.55 | 94.20 | 92.01 | 72.35 | 76.60 | 95.34 | 95.61 |
| Quinalphos | 95.57 | 35.40 | 107.62 | 75.88 | 83.66 | 79.32 | 80.77 | 76.09 | 79.15 | 83.62 | 95.75 | 80.14 |
| Quizalofop ethyl | 84.30 | - | 103.57 | 89.17 | 75.64 | 78.24 | 83.56 | 75.91 | 76.59 | 89.37 | 97.68 | 86.90 |
| Simazine | 92.96 | 133.90 | 108.50 | 70.71 | 76.49 | 73.62 | 64.96 | 73.87 | 81.49 | 78.04 | 81.01 | 71.15 |
| Tebuconazole | 95.92 | 1.79 | 122.61 | 119.45 | 112.40 | 108.40 | 101.95 | 102.25 | 109.97 | 111.48 | 128.75 | 118.72 |
| Temephos | 82.93 | 130.77 | 96.84 | 81.99 | 72.91 | 73.18 | 68.16 | 78.99 | 75.49 | 76.50 | 88.00 | 81.25 |
| Tetramethrin | 87.74 | 100.55 | 95.76 | 71.36 | 66.53 | 70.84 | 68.20 | 73.57 | 73.26 | 78.00 | 86.91 | 82.14 |
| Thiacloprid | 96.43 | 20.74 | 111.83 | 73.25 | 86.76 | 86.80 | 83.42 | 81.90 | 87.62 | 86.17 | 90.16 | 79.72 |
| Thiamethoxam | 130.84 | 53.39 | 136.99 | 77.97 | 116.12 | 119.49 | 117.24 | 109.76 | 116.96 | 116.04 | 118.77 | 104.33 |
| Triasulfuron | 12.53 | 108.30 | 14.41 | 81.81 | 14.76 | 13.87 | 4.39 | 71.91 | 26.92 | 27.51 | 9.25 | 75.58 |
| Tricyclazole | 73.94 | 125.89 | 109.01 | 93.49 | 89.01 | 83.64 | 80.75 | 81.12 | 77.25 | 74.37 | 87.40 | 81.41 |
| Triadimefon | 76.65 | 118.92 | 109.32 | 111.25 | 102.17 | 100.29 | 98.97 | 96.99 | 77.53 | 81.15 | 99.11 | 89.59 |
| **Pesticide recovery** |  |  |  |  |  |  |  |  |  |  |  |  |
| <70% | 23 | 10 | 10 | 14 | 19 | 20 | 24 | **7** | 24 | 17 | 13 | 11 |
| 70%-120% | **77** | **83** | **86** | **89** | **82** | **82** | **77** | **95** | **76** | **83** | **86** | **91** |
| >120% | 2 | 10 | 6 | 0 | 1 | 0 | 0 | 0 | 1 | 2 | 2 | 1 |
| Not detected | 1 | 0 | 1 | 0 | 1 | 1 | 2 | 1 | 2 | 1 | 2 | 0 |

ME1= Original QuEChERS extraction method, ME2= Citrate QuEChERS extraction method, ME3= Acetate QuEChERS extraction method, MC= Cleanup method, where, (MC-A) = 25 mg C-18 + 50 mg PSA + 150 mg anhydrous MgSO_4_, (MC-B) = 50 mg PSA + 150 mg anhydrous MgSO_4_, (MC-C) =100 mg PSA + 150 mg anhydrous MgSO_4_ and (MC-D) = only 150 mg anhydrous MgSO_4_.

**Table S3. Recovery percentage of fortified pesticides at 1 µg g^-1^ in mango fruit drink sample by QuEChERS citrate extraction and clean-up combinations upon varied levels of dilution**

|  | **ME2-MC-A** | | | | **ME2-MC-B** | | | | **ME2-MC-C** | | | | **ME2-MC-D** | | | |
| --- | --- | --- | --- | --- | --- | --- | --- | --- | --- | --- | --- | --- | --- | --- | --- | --- |
| **Pesticide** | **5 mL** | **4 mL** | **2 mL** | **0 mL** | **5 mL** | **4 mL** | **2 mL** | **0 mL** | **5 mL** | **4 mL** | **2 mL** | **0 mL** | **5 mL** | **4 mL** | **2 mL** | **0 mL** |
| Alpha-Cypermethin | 76.45 | 83.41 | 90.03 | 89.94 | 76.58 | 70.68 | 74.83 | 111.31 | 74.90 | 97.36 | 102.50 | 110.95 | 102.14 | 88.85 | 112.06 | 101.93 |
| Allethrin | 92.44 | 90.50 | 96.79 | 98.88 | 81.42 | 79.48 | 82.54 | 113.29 | 70.06 | 117.44 | 87.92 | 112.90 | 112.03 | 97.78 | 102.48 | 114.81 |
| Anilophos | 83.26 | 87.22 | 89.57 | 88.49 | 82.91 | 177.99 | 77.11 | 113.80 | 78.71 | 113.80 | 100.67 | 104.32 | 111.27 | 99.08 | 107.27 | 101.90 |
| Atrazine | 88.10 | 97.31 | 96.78 | 96.33 | 97.10 | 91.25 | 89.47 | 116.81 | 71.22 | 104.81 | 93.67 | 102.39 | 111.57 | 86.91 | 101.93 | 102.56 |
| Azimsulfuron | 49.76 | 71.61 | 72.56 | 71.55 | 62.83 | 67.07 | 67.59 | 63.79 | 46.72 | 21.47 | 38.62 | 39.88 | 111.59 | 48.98 | 103.23 | 88.87 |
| Azoxystrobin | 79.83 | 92.38 | 92.36 | 92.71 | 86.27 | 83.79 | 83.91 | 105.68 | 73.57 | 105.85 | 87.73 | 94.27 | 112.73 | 77.21 | 99.98 | 97.72 |
| Bensulfuron-methyl | 39.91 | 77.67 | 80.55 | 82.09 | 71.67 | 73.13 | 74.03 | 52.68 | 33.39 | 17.55 | 33.78 | 25.84 | 105.91 | 77.48 | 90.21 | 85.80 |
| Bentazone | - | 19.22 | 26.86 | 34.07 | 22.47 | 34.83 | 66.51 | - | - | - | - | - | 21.55 | 53.34 | 88.86 | 97.58 |
| Bifenthrin | 74.29 | 97.22 | 95.51 | 91.84 | 92.86 | 86.61 | 92.81 | 102.91 | 50.53 | 51.46 | 94.91 | 99.22 | 93.67 | 77.66 | 101.90 | 88.65 |
| Bispyribac sodium | - | 22.33 | 26.52 | 29.83 | 21.57 | 27.16 | 41.33 | 6.71 | - | - | 87.50 | - | 29.40 | 10.88 | 66.81 | 78.83 |
| Bitertenol | 91.78 | 75.74 | 79.25 | 78.06 | 59.93 | 65.97 | 66.45 | 115.17 | 62.42 | 54.47 | 66.53 | 54.39 | 111.76 | 108.90 | 103.34 | 110.27 |
| Bromodiolone | - | 49.90 | 47.44 | 48.86 | 48.84 | 55.29 | 58.45 | - | - | - | - | - | - | - | - | - |
| Buprofezin | 78.23 | 91.56 | 93.27 | 92.36 | 83.78 | 80.54 | 80.90 | 101.25 | 73.21 | 105.94 | 95.88 | 96.66 | 108.76 | 80.24 | 105.77 | 94.69 |
| Butachlor | 90.24 | 101.89 | 107.44 | 103.62 | 88.23 | 84.69 | 89.06 | 107.11 | 117.06 | 115.44 | 98.15 | 94.64 | 115.26 | 97.93 | 108.78 | 115.41 |
| Carbaryl | 87.47 | 113.77 | 111.59 | 108.73 | 112.59 | 108.27 | 104.14 | 102.83 | 71.26 | 98.59 | 104.70 | 98.02 | 105.66 | 80.06 | 104.26 | 101.12 |
| Carbendazim | 198.00 | 94.42 | 93.90 | 95.56 | 91.62 | 87.66 | 86.50 | 103.06 | 78.92 | 110.79 | 95.02 | 88.75 | 116.93 | 71.73 | 103.61 | 96.82 |
| Carbofuran | 77.97 | 100.99 | 101.70 | 99.52 | 99.81 | 94.11 | 91.92 | 110.77 | 72.91 | 109.06 | 95.71 | 101.94 | 106.11 | 88.04 | 93.93 | 100.64 |
| Carboxin | 84.26 | 86.84 | 87.38 | 86.35 | 80.71 | 76.29 | 75.75 | 107.57 | 57.57 | 55.11 | 86.75 | 97.67 | 100.90 | 79.56 | 95.29 | 97.45 |
| Carfentrazone ethyl | 89.23 | 75.41 | 78.34 | 79.77 | 73.28 | 70.85 | 70.48 | 117.01 | 106.40 | 116.66 | 109.57 | 107.84 | 119.03 | 118.24 | 109.39 | 105.38 |
| Carpropamid | 77.29 | 100.33 | 100.59 | 102.72 | 92.91 | 87.00 | 89.13 | 119.73 | 91.92 | 111.27 | 103.28 | 113.58 | 116.73 | 109.09 | 113.02 | 117.41 |
| Chlorantraliprole | 75.53 | 77.85 | 80.65 | 80.75 | 74.93 | 71.47 | 70.30 | 105.97 | 61.95 | 56.49 | 97.64 | 91.86 | 104.85 | 92.67 | 102.87 | 88.69 |
| Chlorpyriphos | 192.35 | 93.32 | 99.45 | 98.96 | 90.41 | 83.99 | 84.25 | 209.34 | 81.83 | 105.63 | 99.73 | 207.50 | 107.80 | 76.64 | 104.80 | 109.13 |
| Chlorpyriphos-methyl | 72.11 | 94.57 | 108.83 | 84.98 | 92.13 | 81.22 | 83.65 | 94.81 | 47.32 | 36.28 | 79.48 | 88.15 | 96.43 | 77.74 | 100.27 | 91.10 |
| Chlothianidine | 93.69 | 99.52 | 101.71 | 101.83 | 91.94 | 89.51 | 89.54 | 112.80 | 109.67 | 116.27 | 112.41 | 109.05 | 118.12 | 119.98 | 98.90 | 106.45 |
| Clodinafop-propargyl | 85.40 | 93.52 | 95.95 | 95.89 | 90.16 | 85.63 | 83.79 | 114.25 | 80.96 | 114.22 | 118.58 | 104.92 | 115.16 | 96.76 | 118.11 | 107.02 |
| Clomazone | 84.80 | 94.17 | 93.64 | 93.52 | 91.07 | 87.32 | 85.16 | 108.55 | 70.59 | 108.89 | 105.98 | 104.87 | 112.03 | 86.63 | 109.04 | 100.93 |
| Cyhalofop butyl | 62.87 | - | - | - | - | - | - | 103.19 | 81.61 | 114.03 | 89.40 | 95.58 | 104.74 | 98.51 | 89.41 | 90.05 |
| Cyhalothrin-lambda | 76.19 | 86.20 | 90.34 | 85.44 | 80.31 | 70.36 | 73.25 | 102.67 | - | - | 87.31 | 95.63 | 99.54 | 80.33 | 91.73 | 98.77 |
| Cymoxanil | 93.46 | 150.09 | 135.96 | 130.47 | 148.60 | 147.45 | 113.41 | 119.09 | 78.48 | 114.65 | 73.73 | 107.92 | 118.01 | 99.80 | 86.53 | 114.85 |
| Cyphenothrin | 87.19 | 83.04 | 84.03 | 84.87 | 75.25 | 70.50 | 72.22 | 106.78 | 46.82 | 94.88 | 90.46 | 100.38 | 96.51 | 78.69 | 102.84 | 99.66 |
| Diafenthiuron | 85.73 | 52.59 | 58.70 | 82.59 | 61.37 | 87.49 | 82.40 | 110.58 | - | - | 113.74 | 96.54 | 97.80 | 73.53 | 117.87 | 82.91 |
| Diazinone | 70.11 | 93.34 | 93.88 | 93.44 | 89.50 | 85.75 | 84.70 | 91.02 | 55.89 | 9.90 | 90.61 | 84.09 | 101.16 | 81.26 | 100.20 | 93.98 |
| Diclofop-Methyl | 113.12 | 110.11 | 117.94 | 107.33 | 99.92 | 96.21 | 102.95 | 86.24 | 86.20 | 115.16 | 89.20 | 84.97 | 81.52 | 116.73 | 97.76 | 111.55 |
| Difenoconazole | 82.01 | 84.16 | 85.87 | 87.49 | 78.16 | 75.75 | 74.35 | 114.42 | 78.36 | 113.33 | 107.05 | 112.31 | 118.06 | 97.66 | 112.10 | 103.64 |
| Diflubenzuron | 95.16 | 97.23 | 99.33 | 97.18 | 87.55 | 92.07 | 87.81 | 119.25 | 96.97 | 117.04 | 118.17 | 119.55 | 117.01 | 98.74 | 102.11 | 113.05 |
| Dimethoate | 85.43 | 97.39 | 97.97 | 98.06 | 92.23 | 88.43 | 89.95 | 105.85 | 72.56 | 104.43 | 80.11 | 96.44 | 109.10 | 80.27 | 89.52 | 93.85 |
| Dimethomorph | 82.75 | 74.20 | 77.61 | 76.78 | 54.62 | 64.72 | 73.73 | 107.95 | 73.48 | 103.88 | 61.67 | 63.74 | 112.10 | 80.09 | 105.35 | 103.55 |
| Dinotefuron | 82.23 | 74.95 | 71.71 | 78.78 | 75.91 | 73.27 | 72.45 | 104.69 | 75.55 | 106.42 | 115.34 | 94.39 | 104.99 | 82.70 | 119.46 | 103.17 |
| Edifenphos | 89.23 | 98.83 | 99.84 | 98.84 | 94.65 | 90.24 | 89.81 | 115.52 | 50.61 | 59.39 | 78.41 | 103.49 | 115.35 | 112.56 | 88.65 | 105.22 |
| Ethiprole | 113.79 | 94.76 | 100.73 | 96.65 | 86.82 | 84.34 | 83.59 | 111.71 | 116.94 | 114.71 | 92.97 | 118.02 | 117.84 | 109.98 | 100.10 | 116.56 |
| Ethoxysulfuron | 38.65 | 42.13 | 45.13 | 47.53 | 37.53 | 42.06 | 51.60 | 50.53 | 34.45 | 14.37 | 64.44 | 26.00 | 49.15 | 66.30 | 97.53 | 75.86 |
| Fenamidone | 84.55 | 88.35 | 89.90 | 89.32 | 84.19 | 77.16 | 77.94 | 112.04 | 74.35 | 105.23 | 99.85 | 102.49 | 107.56 | 91.56 | 101.35 | 95.99 |
| Fenarimol | 93.39 | 75.69 | 77.29 | 83.60 | 64.89 | 66.37 | 70.73 | 119.93 | 58.26 | 55.05 | 58.40 | 44.78 | 114.31 | 110.71 | 99.18 | 115.99 |
| Fenazaquin | 70.26 | 92.69 | 93.42 | 93.89 | 89.42 | 80.65 | 84.25 | 96.18 | 55.60 | 59.42 | 108.74 | 87.45 | 97.76 | 76.43 | 107.77 | 86.56 |
| Fenoxaprop-p-ethyl | 77.33 | 80.94 | 81.04 | 78.64 | 79.67 | 70.53 | 74.68 | 103.36 | 96.87 | 104.50 | 94.76 | 93.13 | 107.09 | 96.82 | 106.33 | 100.77 |
| Fenpyroximate | 79.53 | 86.15 | 87.66 | 89.41 | 78.74 | 76.00 | 76.80 | 108.61 | 74.15 | 102.16 | 95.25 | 97.42 | 108.32 | 99.83 | 110.98 | 94.64 |
| Fenvalerate | 79.33 | 81.97 | 97.27 | 97.50 | 60.77 | 64.33 | 76.55 | 95.44 | - | 106.01 | 45.03 | 52.82 | 101.65 | 72.86 | 100.43 | 98.03 |
| Fipronil | - | 60.82 | 24.40 | 34.14 | 95.80 | 117.30 | 75.83 | 49.49 | - | 114.52 | 83.48 | 83.64 | 53.37 | 57.94 | 50.40 | 66.83 |
| Fluazifop-p-butyl | 74.65 | 81.37 | 82.24 | 81.74 | 79.60 | 74.23 | 74.82 | 96.38 | 59.45 | 57.91 | 48.50 | 90.00 | 101.35 | 93.20 | 100.11 | 95.41 |
| Flubendiamide | 106.82 | 67.20 | 70.68 | 74.00 | 60.59 | 57.54 | 57.37 | 117.73 | - | - | 90.46 | 108.71 | 49.54 | 90.94 | 103.56 | 109.15 |
| Flucythrinate | 110.10 | 86.27 | 87.43 | 88.16 | 70.97 | 70.72 | 71.36 | 118.79 | 114.33 | 111.72 | 96.00 | 128.55 | 62.48 | 113.76 | 114.61 | 116.84 |
| Flufenacet | 83.50 | 95.91 | 96.90 | 95.33 | 96.14 | 88.56 | 89.33 | 107.76 | 75.22 | 109.07 | 96.30 | 98.11 | 113.70 | 83.65 | 109.86 | 104.97 |
| Flufenoxuron | 88.07 | 72.90 | 79.14 | 82.73 | 63.96 | 62.29 | 64.31 | 115.39 | 62.73 | 36.44 | 64.38 | 62.62 | 119.55 | 95.61 | 106.19 | 101.76 |
| Flusilazole | 81.13 | 89.74 | 89.43 | 92.15 | 87.42 | 80.56 | 81.92 | 109.76 | 72.23 | 104.78 | 87.01 | 98.12 | 108.91 | 85.05 | 98.63 | 100.40 |
| Forchlofenuron | 80.08 | 99.22 | 99.60 | 98.40 | 95.53 | 90.92 | 97.04 | 106.80 | 75.54 | 89.77 | 89.79 | 87.59 | 110.34 | 96.76 | 97.69 | 98.03 |
| Halosulfuron-methyl | 59.52 | 42.88 | 47.15 | 51.77 | 37.74 | 43.39 | 58.55 | 82.66 | 40.85 | 32.57 | 51.66 | 51.47 | 107.80 | 77.16 | 79.27 | 94.19 |
| Haloxyfop methyl | 79.61 | 112.39 | 110.97 | 110.16 | 103.21 | 96.32 | 99.28 | 102.40 | 78.09 | 103.92 | 81.92 | 95.64 | 109.10 | 86.30 | 89.93 | 99.04 |
| Hexaconazole | 87.96 | 93.89 | 93.72 | 92.20 | 88.26 | 84.09 | 86.35 | 114.47 | 80.18 | 118.60 | 99.35 | 106.61 | 115.73 | 103.37 | 98.62 | 115.96 |
| Hexythiazox | 85.77 | 83.74 | 85.64 | 90.33 | 72.65 | 72.92 | 70.77 | 117.44 | 72.41 | 103.39 | 102.39 | 112.86 | 111.34 | 82.21 | 105.19 | 97.05 |
| Imazamox | 111.41 | 27.63 | 28.84 | 21.87 | 23.13 | 22.20 | 20.22 | 76.69 | - | 104.01 | 62.93 | 14.56 | 96.54 | 65.78 | 53.50 | 78.66 |
| Imidacloprid | 80.77 | 88.62 | 94.87 | 94.18 | 77.08 | 78.04 | 74.25 | 106.58 | 94.26 | 106.42 | 118.16 | 98.81 | 110.30 | 94.88 | 88.47 | 100.83 |
| Indoxacarb | 80.80 | 80.04 | 80.13 | 82.19 | 71.20 | 71.66 | 72.60 | 103.65 | 91.57 | 114.08 | 102.32 | 99.34 | 116.37 | 93.25 | 110.64 | 99.86 |
| Iprovalicarb | 84.46 | 87.04 | 88.54 | 88.84 | 85.12 | 78.56 | 77.57 | 107.16 | 54.26 | 26.73 | 99.11 | 98.63 | 110.31 | 76.05 | 105.45 | 100.74 |
| Isopropalin | 88.76 | 99.15 | 95.29 | 92.60 | 90.31 | 84.29 | 87.09 | 92.85 | 48.71 | 47.70 | 86.30 | 84.21 | 86.25 | 71.51 | 95.01 | 87.65 |
| Isoprothiolane | 76.03 | 90.87 | 92.01 | 91.29 | 89.41 | 83.00 | 81.73 | 104.56 | 77.35 | 104.53 | 26.68 | 97.70 | 102.15 | 77.49 | 100.12 | 93.10 |
| Kresoxim methyl | 78.21 | 91.06 | 91.60 | 91.58 | 87.04 | 78.94 | 83.30 | 105.39 | 84.92 | 107.74 | 119.04 | 99.46 | 110.14 | 99.13 | 115.62 | 102.14 |
| Lactofen | 89.48 | 81.67 | 84.79 | 86.45 | 70.95 | 71.05 | 70.22 | 113.50 | 90.32 | 118.83 | 79.53 | 115.81 | 112.65 | 114.53 | 108.53 | 116.65 |
| Malathion | 51.66 | 86.02 | 86.16 | 85.73 | 79.70 | 78.46 | 75.37 | 110.37 | 98.65 | 100.85 | 99.75 | 90.19 | 75.36 | 89.18 | 115.80 | 89.96 |
| Metaflumizone | 104.14 | 75.38 | 81.07 | 76.41 | 65.84 | 65.11 | 64.42 | 99.34 | - | 60.88 | 8.89 | 44.46 | 107.45 | 87.57 | 99.09 | 101.05 |
| Metalaxyl | 78.22 | 96.52 | 96.45 | 95.97 | 94.49 | 88.45 | 88.54 | 106.84 | 79.66 | 106.18 | 83.43 | 96.87 | 105.79 | 88.95 | 94.14 | 102.48 |
| Methabenzthiazuron | 82.90 | 102.01 | 100.28 | 100.34 | 102.50 | 95.47 | 93.67 | 109.94 | 77.34 | 107.33 | 87.42 | 101.01 | 111.06 | 78.52 | 113.72 | 96.28 |
| Methomyl | 73.60 | 93.73 | 92.62 | 91.71 | 89.51 | 84.45 | 83.91 | 98.13 | 76.02 | 101.09 | 87.88 | 94.24 | 99.54 | 64.84 | 60.82 | 91.71 |
| Metsulfuron-methyl | 38.08 | 115.01 | 119.19 | 116.12 | 101.87 | 108.94 | 108.70 | 54.06 | 31.51 | 13.61 | 58.06 | 26.53 | 113.05 | 74.66 | 108.59 | 96.27 |
| Myclobutanil | 94.40 | 88.88 | 93.95 | 92.27 | 87.53 | 82.40 | 81.08 | 115.57 | 84.85 | 117.75 | 98.43 | 106.67 | 119.83 | 104.78 | 99.21 | 112.62 |
| Oxycarboxin | 78.29 | 100.55 | 99.98 | 100.31 | 97.71 | 91.00 | 92.03 | 101.04 | 59.15 | 66.02 | 87.38 | 79.42 | 116.63 | 89.99 | 93.47 | 105.83 |
| Penconazole | 94.08 | 99.55 | 99.90 | 99.46 | 96.32 | 90.22 | 90.70 | 115.75 | 110.88 | 118.44 | 91.66 | 119.43 | 115.28 | 104.84 | 106.42 | 116.10 |
| Pencycuron | 77.89 | 91.62 | 94.29 | 94.22 | 89.05 | 80.84 | 83.53 | 106.60 | 70.06 | 105.45 | 91.09 | 95.91 | 107.81 | 86.54 | 102.95 | 95.61 |
| Pendimethalin | 81.33 | 107.48 | 118.85 | 112.80 | 103.44 | 88.16 | 100.59 | 97.98 | 72.62 | 109.75 | 112.45 | 92.45 | 102.55 | 75.89 | 117.15 | 108.12 |
| Permethrin | 82.54 | 94.99 | 95.69 | 95.00 | 90.88 | 82.59 | 84.93 | 108.45 | 40.36 | 57.05 | 93.21 | 103.01 | 90.73 | 76.35 | 105.80 | 90.05 |
| Phorate | 72.16 | 88.59 | 85.44 | 89.11 | 75.32 | 73.31 | 74.06 | 95.32 | 61.97 | 50.97 | 98.59 | 88.60 | 95.89 | 77.99 | 105.56 | 94.56 |
| Phosalone | 80.11 | 88.07 | 90.75 | 89.81 | 80.61 | 77.69 | 78.78 | 109.26 | 88.82 | 112.69 | 89.44 | 104.80 | 109.56 | 99.25 | 96.97 | 109.08 |
| Phosphamidon | 78.63 | 89.50 | 90.38 | 89.76 | 83.57 | 79.05 | 79.67 | 101.61 | 57.07 | 49.66 | 47.02 | 88.84 | 99.21 | 72.15 | 97.55 | 96.57 |
| Pretilachlor | 80.83 | 83.28 | 85.31 | 83.27 | 81.60 | 74.74 | 76.66 | 99.55 | 75.64 | 105.29 | 77.16 | 95.94 | 113.28 | 86.16 | 84.51 | 101.61 |
| Profenophos | 83.40 | 79.99 | 77.48 | 79.05 | 79.44 | 76.30 | 74.74 | 118.03 | 91.14 | 109.86 | 111.95 | 104.32 | 117.05 | 104.48 | 117.99 | 107.47 |
| Propachlor | 86.91 | 93.58 | 95.36 | 93.52 | 90.97 | 85.84 | 85.40 | 118.18 | 71.40 | 111.65 | 111.49 | 110.92 | 113.18 | 89.55 | 116.11 | 101.25 |
| Propanil | - | 100.10 | 98.19 | 95.70 | 102.18 | 97.22 | 92.33 | - | 35.20 | - | 93.92 | - | - | - | 112.69 | - |
| Propazine | 90.02 | 99.89 | 99.94 | 98.70 | 97.86 | 93.34 | 92.23 | 118.02 | 59.37 | 50.66 | 114.59 | 109.35 | 110.56 | 82.32 | 113.12 | 98.41 |
| Propiconazole | 85.90 | 91.74 | 94.58 | 94.33 | 87.31 | 83.42 | 82.63 | 109.18 | 89.73 | 66.34 | 113.66 | 103.84 | 116.79 | 97.87 | 113.06 | 104.94 |
| Propoxur | 78.87 | 88.46 | 88.74 | 89.23 | 84.95 | 80.45 | 79.13 | 99.93 | 71.77 | 107.71 | 111.23 | 92.64 | 101.91 | 78.17 | 119.16 | 101.03 |
| Pyraclostrobin | 75.87 | 80.54 | 82.51 | 83.84 | 76.04 | 71.31 | 73.01 | 104.28 | 72.22 | 106.94 | 89.88 | 92.74 | 101.10 | 81.18 | 101.02 | 101.36 |
| Pyrazosulfuron-ethyl | 62.76 | 62.60 | 76.18 | 73.29 | 62.54 | 63.44 | 66.25 | 82.53 | 49.40 | 30.96 | 54.81 | 56.81 | 105.95 | 77.41 | 102.68 | 95.31 |
| Pyriproxyfen | 87.17 | 90.78 | 95.31 | 96.16 | 85.65 | 80.53 | 82.05 | 98.92 | 80.57 | 91.32 | 91.43 | 84.73 | 94.45 | 83.34 | 97.43 | 91.06 |
| Quinalphos | 93.53 | 87.56 | 90.43 | 90.14 | 84.66 | 78.87 | 78.55 | 114.76 | 104.64 | 110.41 | 83.85 | 112.52 | 102.88 | 111.44 | 94.86 | 118.47 |
| Quizalofop ethyl | 87.79 | 49.42 | 53.95 | 51.09 | 52.94 | 50.29 | 47.88 | 105.08 | 102.33 | 115.73 | 0.00 | 51.02 | 115.57 | 119.14 | - | 108.33 |
| Simazine | 86.29 | 93.99 | 95.62 | 93.18 | 89.60 | 85.87 | 85.32 | 104.79 | 57.93 | 61.52 | 105.23 | 105.37 | 105.63 | 76.41 | 114.54 | 92.05 |
| Tebuconazole | 103.65 | 86.75 | 88.53 | 88.32 | 79.69 | 77.73 | 76.45 | 119.71 | 111.55 | 115.43 | 1.15 | 116.05 | 119.27 | 111.74 | 96.18 | 118.49 |
| Temephos | 72.07 | 81.45 | 88.31 | 95.10 | 74.10 | 72.83 | 71.16 | 101.76 | 82.06 | 113.41 | 102.06 | 95.53 | 100.09 | 97.95 | 108.19 | 109.34 |
| Tetramethrin | 88.51 | 98.02 | 107.37 | 108.05 | 90.87 | 84.95 | 86.04 | 95.64 | 116.73 | 100.40 | 90.69 | 96.26 | 97.97 | 95.76 | 98.43 | 113.63 |
| Thiacloprid | 85.57 | 98.03 | 97.47 | 96.91 | 93.96 | 89.55 | 89.77 | 113.06 | 90.19 | 112.00 | 18.44 | 101.01 | 112.46 | 96.40 | 98.60 | 104.03 |
| Thiamethoxam | 99.27 | 79.51 | 80.00 | 82.57 | 72.10 | 70.71 | 72.72 | 115.30 | 98.96 | 106.46 | 60.62 | 110.44 | 109.51 | 102.53 | 92.21 | 107.00 |
| Triasulfuron | 31.32 | 78.94 | 80.83 | 81.60 | 66.89 | 70.28 | 76.91 | 47.90 | 28.88 | 8.71 | 64.81 | 21.09 | 107.79 | 87.89 | 91.86 | 87.26 |
| Tricyclazole | 93.82 | 98.17 | 96.76 | 96.63 | 95.18 | 90.51 | 91.87 | 114.09 | 74.26 | 110.79 | 112.18 | 111.00 | 107.55 | 83.47 | 113.47 | 99.75 |
| Triadimefon | 89.06 | 99.98 | 98.86 | 98.79 | 92.94 | 91.11 | 88.24 | 119.34 | 73.47 | 113.85 | - | 108.15 | 119.30 | 90.51 | - | 103.64 |
| **Pesticide recovery** |  | | | | | | | | | | | | | | | |
| <70% | 10 | 11 | 9 | 8 | 18 | 17 | 13 | **7** | 28 | 29 | 21 | 16 | 6 | 7 | 4 | 1 |
| 70%-120% | **86** | **90** | **92** | **93** | **83** | **84** | **89** | **92** | **65** | **68** | **79** | **82** | **95** | **94** | **96** | **100** |
| >120% | 2 | 1 | 1 | 1 | 1 | 1 | 0 | 1 | 0 | 0 | 0 | 2 | 0 | 0 | 0 | 0 |
| Not detected | 5 | 1 | 1 | 1 | 1 | 1 | 1 | 3 | 10 | 7 | 3 | 4 | 2 | 2 | 3 | 2 |

**Where, ME2- Citrate QueCHERS with clean-up combination, MC-A = 25 mg C-18 + 50 mg PSA + 150 mg anhydrous MgSO_4_, MC-B = 50 mg PSA + 150 mg anhydrous MgSO_4_, MC-C =100 mg PSA + 150 mg anhydrous MgSO_4_ and MC-D = only 150 mg anhydrous MgSO_4_.**

**Table S4. Validation of developed method for recovery (solvent and matrix match standard) at different fortification levels in mango fruit drink.**

|  |  | **0.1 µg g^-1^** | | | | **0.05 µg g^-1^** | | | | **0.01 µg g^-1^** | | | |  | | | | |
| --- | --- | --- | --- | --- | --- | --- | --- | --- | --- | --- | --- | --- | --- | --- | --- | --- | --- | --- |
| **Sl.**  **No.** | **Pesticides** | **RSS**  ***** | **RMM**  **#** | **RSD**  **©** | **Hor**  **Rat●** | **RSS**  ***** | **RMM**  **#** | **RSD**  **©** | **Hor**  **Rat●** | **RSS**  ***** | **RMM**  **#** | **RSD**  **©** | **Hor**  **Rat●** | **Specificity** | **LOQ^&^** | **ME $ @ LOQ** | **GU** | **U**  **(%)** |
| 1 | Alpha-Cypermethin | 94.82 | 83.72 | 8.16 | 0.36 | 104.19 | 85.69 | 12.95 | 0.52 | 79.50 | 92.05 | 14.48 | 0.46 | 6.42 | 0.01 | 15.79 | 0.0028 | 18.09 |
| 2 | Allethrin | 83.60 | 81.54 | 17.17 | 0.77 | 96.84 | 90.00 | 4.68 | 0.19 | 80.30 | 103.11 | 13.13 | 0.41 | 17.78 | 0.01 | 28.41 | 0.0020 | 16.68 |
| 3 | Anilophos | 75.94 | 73.66 | 14.50 | 0.65 | 95.09 | 86.53 | 2.67 | 0.11 | 93.05 | 98.45 | 8.73 | 0.28 | 1.71 | 0.01 | 5.81 | 0.0025 | 12.16 |
| 4 | Atrazine | 75.84 | 81.11 | 5.47 | 0.24 | 108.57 | 110.36 | 16.61 | 0.67 | 84.21 | 101.86 | 10.33 | 0.33 | 11.04 | 0.01 | 20.95 | 0.0026 | 13.32 |
| 5 | Azimsulfuron | 92.22 | 101.31 | 19.13 | 0.85 | 78.72 | 81.86 | 12.37 | 0.50 | 80.33 | 114.81 | 9.59 | 0.30 | 0.00 | 0.01 | 42.92 | 0.0021 | 12.08 |
| 6 | Azoxystrobin | 87.07 | 89.99 | 19.37 | 0.86 | 80.56 | 80.69 | 9.98 | 0.40 | 79.76 | 80.38 | 12.22 | 0.39 | 1.59 | 0.01 | 0.78 | 0.0020 | 16.04 |
| 7 | Bensulfuron-methyl | 51.40 | 54.30 | 17.53 | 0.78 | 47.01 | 46.80 | 10.28 | 0.41 | 33.78 | 48.85 | 9.70 | 0.31 | 0.09 | 1 | - | 0.0472 | 4.72 |
| 8 | Bentazone | 43.06 | 56.23 | 8.37 | 0.37 | - | - | - | - | - | - | - | - | 3.16 | 0.1 | 30.58 | 0.0141 | 14.12 |
| 9 | Bifenthrin | 74.96 | 77.74 | 7.48 | 0.33 | 89.80 | 88.09 | 8.38 | 0.34 | 74.40 | 87.75 | 9.72 | 0.31 | 5.53 | 0.01 | 17.94 | 0.0015 | 15.28 |
| 10 | Bispyribac sodium | 36.34 | 31.28 | 20.36 | 0.91 | 8.60 | 7.64 | 9.05 | 0.36 | - | - | - | - | 0.00 | 1 | - | 0.0639 | 6.39 |
| 11 | Bitertenol | 118.65 | 99.66 | 17.73 | 0.79 | 112.12 | 87.75 | 6.16 | 0.25 | 74.54 | 99.76 | 11.51 | 0.36 | 9.91 | 0.01 | 33.83 | 0.0022 | 17.27 |
| 12 | Bromodiolone | - | - | - | - | - | - | - | - | - | - | - | - | 0.00 | 1 | - | 0.2019 | 19.01 |
| 13 | Buprofezin | 86.82 | 83.69 | 16.60 | 0.74 | 96.94 | 86.46 | 7.74 | 0.31 | 91.92 | 107.39 | 16.31 | 0.51 | 15.00 | 0.01 | 16.83 | 0.0020 | 19.58 |
| 14 | Butachlor | 85.65 | 79.32 | 6.62 | 0.30 | 90.55 | 78.84 | 10.70 | 0.43 | 80.69 | 95.77 | 13.74 | 0.43 | 24.01 | 0.01 | 18.69 | 0.0019 | 19.08 |
| 15 | Carbaryl | 76.28 | 80.82 | 5.87 | 0.26 | 84.38 | 87.32 | 4.74 | 0.19 | 91.69 | 101.26 | 11.71 | 0.37 | 12.94 | 0.01 | 10.43 | 0.0022 | 15.55 |
| 16 | Carbendazim | 70.37 | 79.39 | 3.86 | 0.17 | 79.88 | 87.31 | 2.96 | 0.12 | 89.03 | 82.89 | 6.27 | 0.20 | 4.61 | 0.01 | -6.89 | 0.0020 | 9.42 |
| 17 | Carbofuran | 75.46 | 84.44 | 19.73 | 0.88 | 84.11 | 90.53 | 3.23 | 0.13 | 90.02 | 97.08 | 15.44 | 0.49 | 1.99 | 0.01 | 7.84 | 0.0023 | 17.99 |
| 18 | Carboxin | 72.51 | 75.80 | 5.74 | 0.26 | 78.49 | 78.80 | 3.51 | 0.14 | 91.59 | 113.57 | 19.56 | 0.62 | 3.52 | 0.01 | 23.88 | 0.0029 | 22.92 |
| 19 | Carfentrazone ethyl | 91.29 | 85.14 | 10.84 | 0.48 | 91.54 | 82.57 | 9.78 | 0.39 | 82.73 | 98.38 | 19.79 | 0.62 | 3.22 | 0.01 | 18.91 | 0.0024 | 23.89 |
| 20 | Carpropamid | 89.46 | 84.08 | 4.75 | 0.21 | 100.77 | 91.92 | 5.66 | 0.23 | 93.81 | 104.26 | 11.08 | 0.35 | 4.89 | 0.01 | 11.15 | 0.0021 | 16.12 |
| 21 | Chlorantraliprole | 73.28 | 79.54 | 7.36 | 0.33 | 88.43 | 91.05 | 12.62 | 0.51 | 78.74 | 99.29 | 8.35 | 0.26 | 3.02 | 0.01 | 26.10 | 0.0012 | 12.32 |
| 22 | Chlorpyriphos | 106.78 | 98.55 | 12.31 | 0.55 | 112.41 | 92.69 | 9.84 | 0.40 | 87.51 | 86.57 | 13.19 | 0.42 | 14.36 | 0.01 | -1.08 | 0.0019 | 18.86 |
| 23 | Chlorpyriphos-methyl | 90.26 | 82.37 | 18.72 | 0.83 | 100.08 | 85.59 | 10.36 | 0.42 | 58.64 | 93.98 | 16.39 | 0.52 | 27.55 | 0.01 | 60.28 | 0.0028 | 19.42 |
| 24 | Chlothianidine | 78.49 | 86.03 | 6.99 | 0.31 | 82.98 | 83.13 | 5.57 | 0.22 | 79.21 | 116.72 | 9.60 | 0.30 | 1.70 | 0.01 | 47.35 | 0.0017 | 13.14 |
| 25 | Clodinafop-propargyl | 81.24 | 87.29 | 11.54 | 0.51 | 84.44 | 84.36 | 7.42 | 0.30 | 79.91 | 91.08 | 9.25 | 0.29 | 2.38 | 0.01 | 13.98 | 0.0018 | 11.15 |
| 26 | Clomazone | 76.96 | 84.01 | 18.11 | 0.81 | 84.65 | 88.87 | 3.21 | 0.13 | 97.13 | 103.72 | 17.54 | 0.55 | 6.80 | 0.01 | 6.69 | 0.0029 | 20.61 |
| 27 | Cyhalofop butyl | 87.81 | 80.38 | 9.73 | 0.43 | 95.97 | 79.08 | 12.70 | 0.51 | 87.33 | 96.74 | 13.32 | 0.42 | 5.82 | 0.01 | 10.77 | 0.0023 | 15.68 |
| 28 | Cyhalothrin-lambda | 83.14 | 85.38 | 16.13 | 0.72 | 89.30 | 85.85 | 9.40 | 0.38 | 78.31 | 94.23 | 15.97 | 0.50 | 9.26 | 0.01 | 20.32 | 0.0023 | 19.42 |
| 29 | Cymoxanil | 77.16 | 84.20 | 5.38 | 0.24 | 85.76 | 88.60 | 13.18 | 0.53 | 80.54 | 96.83 | 11.16 | 0.35 | 2.69 | 0.01 | 20.22 | 0.0016 | 16.44 |
| 30 | Cyphenothrin | 84.93 | 81.58 | 5.72 | 0.25 | 90.16 | 79.22 | 4.83 | 0.19 | 56.72 | 88.14 | 18.77 | 0.59 | 3.12 | 0.01 | 55.39 | 0.0029 | 22.13 |
| 31 | Diafenthiuron | 86.34 | 81.33 | 13.22 | 0.59 | 108.27 | 97.11 | 7.95 | 0.32 | - | - | - | - | 4.05 | 0.05 | -10.31 | 0.0073 | 14.53 |
| 32 | Diazinone | 86.19 | 77.23 | 3.35 | 0.15 | 98.47 | 85.13 | 4.11 | 0.17 | 87.57 | 82.14 | 13.37 | 0.42 | 14.63 | 0.01 | -6.21 | 0.0026 | 16.25 |
| 33 | Diclofop-Methyl | 80.62 | 81.88 | 13.91 | 0.62 | 86.42 | 91.92 | 8.86 | 0.36 | 108.58 | 98.40 | 8.87 | 0.28 | 19.13 | 0.01 | 6.36 | 0.0012 | 11.57 |
| 34 | Difenoconazole | 101.56 | 78.72 | 0.57 | 0.03 | 111.36 | 82.64 | 8.69 | 0.35 | 102.51 | 78.10 | 10.97 | 0.35 | 2.90 | 0.01 | -23.81 | 0.0022 | 13.90 |
| 35 | Diflubenzuron | 87.56 | 82.96 | 7.46 | 0.33 | 89.66 | 76.37 | 9.41 | 0.38 | 76.48 | 106.67 | 17.99 | 0.57 | 1.56 | 0.01 | 39.47 | 0.0025 | 21.97 |
| 36 | Dimethoate | 71.11 | 82.88 | 15.78 | 0.70 | 80.77 | 88.82 | 9.54 | 0.38 | 77.88 | 94.21 | 9.74 | 0.31 | 0.56 | 0.01 | 20.97 | 0.0013 | 13.18 |
| 37 | Dimethomorph | 84.00 | 82.12 | 7.04 | 0.31 | 87.39 | 79.31 | 8.63 | 0.35 | 74.56 | 97.69 | 7.81 | 0.25 | 1.03 | 0.01 | 31.03 | 0.0025 | 9.59 |
| 38 | Dinotefuron | 76.88 | 96.41 | 11.86 | 0.53 | 80.04 | 86.60 | 10.58 | 0.42 | 72.62 | 112.87 | 11.67 | 0.37 | 3.54 | 0.01 | 55.44 | 0.0018 | 17.57 |
| 39 | Edifenphos | 95.60 | 98.36 | 14.90 | 0.66 | 92.82 | 88.83 | 13.68 | 0.55 | 100.80 | 101.02 | 16.32 | 0.51 | 3.98 | 0.01 | 0.22 | 0.0028 | 19.74 |
| 40 | Ethiprole | 88.46 | 94.52 | 3.15 | 0.14 | 98.51 | 91.68 | 9.23 | 0.37 | 89.74 | 96.41 | 13.51 | 0.42 | 19.10 | 0.01 | 7.43 | 0.0022 | 16.45 |
| 41 | Ethoxysulfuron | 48.05 | 57.06 | 3.91 | 0.17 | 41.59 | 48.32 | 11.42 | 0.46 | 44.91 | 67.88 | 10.24 | 0.32 | 0.00 | 1 | - | 0.1398 | 16.59 |
| 42 | Fenamidone | 86.23 | 79.51 | 19.51 | 0.87 | 85.95 | 76.00 | 8.66 | 0.35 | 75.40 | 111.22 | 18.77 | 0.59 | 3.13 | 0.01 | 47.51 | 0.0028 | 21.88 |
| 43 | Fenarimol | 207.23 | 185.84 | 7.18 | 0.32 | 525.15 | 245.23 | 7.98 | 0.32 | 88.32 | 115.31 | 9.62 | 0.30 | 29.78 | 0.01 | 30.56 | 0.0012 | 11.88 |
| 44 | Fenazaquin | 83.12 | 81.73 | 12.64 | 0.56 | 85.55 | 79.50 | 5.98 | 0.24 | 85.69 | 88.32 | 11.67 | 0.37 | 2.78 | 0.01 | 3.08 | 0.0023 | 16.13 |
| 45 | Fenoxaprop-p-ethyl | 71.07 | 75.69 | 3.37 | 0.15 | 77.27 | 73.79 | 7.68 | 0.31 | 93.16 | 105.58 | 9.68 | 0.31 | 7.43 | 0.01 | 13.33 | 0.0024 | 14.89 |
| 46 | Fenpyroximate | 90.58 | 70.74 | 14.98 | 0.67 | 108.50 | 82.07 | 7.79 | 0.31 | 83.65 | 100.18 | 9.66 | 0.30 | 4.54 | 0.01 | 19.90 | 0.0020 | 13.50 |
| 47 | Fenvalerate | 70.83 | 74.75 | 12.29 | 0.55 | 93.00 | 99.15 | 17.30 | 0.69 | - | - | - | - | 6.83 | 0.05 | 6.60 | 0.0115 | 20.27 |
| 48 | Fipronil | 36.23 | 33.99 | 10.11 | 0.45 | 40.22 | 44.79 | 10.90 | 0.44 | - | - | - | - | 0.00 | 1 | - | 0.0559 | 5.59 |
| 49 | Fluazifop-p-butyl | 86.39 | 80.91 | 8.50 | 0.38 | 93.13 | 84.54 | 4.99 | 0.20 | 72.17 | 92.37 | 18.50 | 0.58 | 2.85 | 0.01 | 28.00 | 0.0025 | 19.59 |
| 50 | Flubendiamide | 71.82 | 119.53 | 9.84 | 0.44 | - | - | - | - | - | - | - | - | 0.00 | 0.1 | 79.60 | 0.0016 | 15.62 |
| 51 | Flucythrinate | 87.41 | 78.54 | 2.40 | 0.11 | 100.30 | 81.80 | 4.00 | 0.16 | - | - | - | - | 0.00 | 0.05 | -18.45 | 0.0030 | 5.92 |
| 52 | Flufenacet | 95.48 | 88.52 | 17.69 | 0.79 | 96.14 | 85.44 | 3.14 | 0.13 | 79.83 | 100.96 | 16.74 | 0.53 | 0.76 | 0.01 | 26.47 | 0.0020 | 19.70 |
| 53 | Flufenoxuron | 88.67 | 79.87 | 7.05 | 0.31 | 95.86 | 83.05 | 9.18 | 0.37 | 75.15 | 93.77 | 18.28 | 0.58 | 0.00 | 0.01 | 24.65 | 0.0022 | 22.19 |
| 54 | Flusilazole | 87.63 | 80.76 | 19.33 | 0.86 | 96.68 | 84.40 | 3.86 | 0.15 | 93.73 | 88.55 | 6.01 | 0.19 | 2.31 | 0.01 | -5.52 | 0.0015 | 8.93 |
| 55 | Forchlofenuron | 82.52 | 82.16 | 9.67 | 0.43 | 93.13 | 87.17 | 20.19 | 0.81 | 77.84 | 96.50 | 12.26 | 0.39 | 1.94 | 0.01 | 23.97 | 0.0015 | 14.91 |
| 56 | Halosulfuron-methyl | 84.85 | 86.16 | 18.40 | 0.82 | 91.66 | 88.77 | 5.91 | 0.24 | 102.49 | 107.85 | 12.97 | 0.41 | 0.00 | 0.01 | 5.23 | 0.0023 | 15.66 |
| 57 | Haloxyfop methyl | 86.19 | 74.47 | 9.76 | 0.43 | 107.60 | 90.34 | 10.70 | 0.43 | 96.72 | 92.59 | 12.70 | 0.40 | 29.77 | 0.01 | -4.26 | 0.0022 | 15.04 |
| 58 | Hexaconazole | 90.12 | 78.12 | 9.58 | 0.43 | 102.71 | 83.83 | 7.44 | 0.30 | 83.75 | 99.71 | 11.64 | 0.37 | 6.84 | 0.01 | 19.19 | 0.0020 | 15.12 |
| 59 | Hexythiazox | 90.72 | 81.44 | 11.86 | 0.53 | 95.04 | 81.90 | 5.78 | 0.23 | 75.21 | 84.41 | 17.60 | 0.56 | 6.04 | 0.01 | 12.24 | 0.0025 | 20.75 |
| 60 | Imazamox | 77.59 | 107.98 | 4.35 | 0.19 | 70.30 | 93.06 | 7.82 | 0.31 | 59.88 | 107.72 | 13.35 | 0.42 | 0.00 | 0.01 | 32.38 | 0.0010 | 14.83 |
| 61 | Imidacloprid | 89.80 | 85.80 | 9.52 | 0.42 | 110.12 | 97.84 | 12.82 | 0.51 | 81.93 | 103.57 | 17.39 | 0.55 | 3.13 | 0.01 | 26.29 | 0.0027 | 21.22 |
| 62 | Indoxacarb | 95.95 | 86.38 | 3.23 | 0.14 | 108.28 | 92.63 | 7.99 | 0.32 | 100.26 | 98.76 | 11.48 | 0.36 | 0.00 | 0.01 | -1.50 | 0.0015 | 15.28 |
| 63 | Iprovalicarb | 88.13 | 82.06 | 9.05 | 0.40 | 98.41 | 87.79 | 12.55 | 0.50 | 88.89 | 96.28 | 9.34 | 0.29 | 1.63 | 0.01 | 8.31 | 0.0016 | 11.71 |
| 64 | Isopropalin | 102.65 | 84.60 | 12.60 | 0.56 | 100.37 | 83.61 | 15.35 | 0.62 | - | - | - | - | 8.46 | 0.05 | -16.79 | 0.0101 | 20.19 |
| 65 | Isoprothiolane | 87.95 | 82.84 | 17.96 | 0.80 | 113.78 | 104.88 | 11.98 | 0.48 | 87.89 | 104.52 | 8.39 | 0.26 | 1.62 | 0.01 | 18.93 | 0.0023 | 11.45 |
| 66 | Kresoxim methyl | 87.21 | 86.47 | 8.66 | 0.39 | 96.21 | 89.91 | 3.77 | 0.15 | 78.03 | 90.51 | 15.86 | 0.50 | 1.83 | 0.01 | 16.00 | 0.0019 | 18.70 |
| 67 | Lactofen | 97.79 | 84.34 | 13.01 | 0.58 | 104.30 | 89.01 | 4.28 | 0.17 | 76.90 | 75.74 | 15.30 | 0.48 | 2.64 | 0.01 | -1.51 | 0.0019 | 18.79 |
| 68 | Malathion | 86.04 | 72.78 | 19.35 | 0.86 | 107.72 | 41.18 | 9.02 | 0.36 | - | - | - | - | 4.93 | 0.05 | -15.40 | 0.0083 | 16.68 |
| 69 | Metaflumizone | 70.10 | 66.14 | 15.24 | 0.68 | 63.80 | 54.08 | 13.03 | 0.52 | - | - | - | - | 0.00 | 0.1 | -5.66 | 0.0195 | 19.54 |
| 70 | Metalaxyl | 79.84 | 85.72 | 11.48 | 0.51 | 85.08 | 86.60 | 7.61 | 0.31 | 92.83 | 99.32 | 3.97 | 0.13 | 2.86 | 0.01 | 6.99 | 0.0011 | 10.89 |
| 71 | Methabenzthiazuron | 79.22 | 81.05 | 11.93 | 0.53 | 87.43 | 85.38 | 11.04 | 0.44 | 87.04 | 102.11 | 4.33 | 0.14 | 1.96 | 0.01 | 17.32 | 0.0018 | 6.38 |
| 72 | Methomyl | 70.87 | 79.70 | 18.55 | 0.83 | 78.48 | 85.25 | 7.56 | 0.30 | 64.37 | 92.02 | 14.26 | 0.45 | 1.56 | 0.01 | 42.95 | 0.0020 | 19.94 |
| 73 | Metsulfuron-methyl | 79.22 | 89.01 | 4.98 | 0.22 | 82.65 | 85.15 | 5.02 | 0.20 | 55.36 | 115.76 | 4.16 | 0.13 | 0.87 | 0.01 | 3.02 | 0.0006 | 9.72 |
| 74 | Myclobutanil | 96.49 | 85.30 | 15.04 | 0.67 | 116.39 | 92.23 | 7.91 | 0.32 | 81.34 | 100.43 | 11.27 | 0.36 | 3.98 | 0.01 | 23.58 | 0.0023 | 14.46 |
| 75 | Oxycarboxin | 77.55 | 83.54 | 18.65 | 0.83 | 86.45 | 87.60 | 6.26 | 0.25 | 89.03 | 106.35 | 7.06 | 0.22 | 1.62 | 0.01 | 19.45 | 0.0021 | 9.19 |
| 76 | Penconazole | 99.46 | 85.13 | 14.68 | 0.65 | 116.89 | 92.96 | 8.01 | 0.32 | 96.19 | 90.87 | 3.37 | 0.11 | 2.79 | 0.01 | -5.53 | 0.0021 | 8.58 |
| 77 | Pencycuron | 85.92 | 75.43 | 6.98 | 0.31 | 98.22 | 82.17 | 2.73 | 0.11 | 91.58 | 96.72 | 3.06 | 0.10 | 3.87 | 0.01 | 5.61 | 0.0019 | 5.43 |
| 78 | Pendimethalin | 84.51 | 86.31 | 4.64 | 0.21 | 94.77 | 91.51 | 3.73 | 0.15 | 115.30 | 98.07 | 19.37 | 0.61 | 2.24 | 0.01 | -3.54 | 0.0006 | 6.12 |
| 79 | Permethrin | 88.54 | 80.52 | 4.62 | 0.21 | 103.26 | 87.41 | 5.68 | 0.23 | 63.29 | 88.78 | 12.85 | 0.41 | 24.38 | 0.01 | 40.26 | 0.0015 | 15.11 |
| 80 | Phorate | 81.96 | 78.62 | 5.55 | 0.25 | 88.49 | 80.92 | 2.59 | 0.10 | 96.60 | 97.51 | 6.69 | 0.21 | 8.02 | 0.01 | 0.94 | 0.0023 | 9.70 |
| 81 | Phosalone | 91.79 | 86.52 | 3.55 | 0.15 | 104.48 | 89.88 | 4.78 | 0.19 | 90.07 | 94.64 | 17.06 | 0.54 | 16.77 | 0.01 | 5.07 | 0.0026 | 20.08 |
| 82 | Phosphamidon | 77.76 | 82.40 | 6.24 | 0.28 | 82.77 | 83.80 | 6.03 | 0.24 | 82.41 | 89.63 | 8.71 | 0.27 | 0.00 | 0.01 | 8.76 | 0.0019 | 13.50 |
| 83 | Pretilachlor | 75.23 | 92.11 | 13.26 | 0.59 | 78.18 | 91.41 | 6.37 | 0.26 | 93.71 | 106.28 | 14.44 | 0.46 | 8.14 | 0.01 | 13.51 | 0.0028 | 17.09 |
| 84 | Profenophos | 88.75 | 83.09 | 9.33 | 0.42 | 94.12 | 85.75 | 6.99 | 0.28 | 83.35 | 94.22 | 17.24 | 0.54 | 4.68 | 0.01 | 13.04 | 0.0020 | 20.50 |
| 85 | Propachlor | 78.79 | 84.91 | 2.90 | 0.13 | 87.28 | 87.85 | 2.62 | 0.11 | 83.74 | 95.78 | 2.75 | 0.09 | 2.17 | 0.01 | 14.38 | 0.0014 | 7.89 |
| 86 | Propanil | 73.54 | 88.02 | 14.35 | 0.64 | - | - | - | - | - | - | - | - | 0.00 | 0.1 | 19.86 | 0.0239 | 19.97 |
| 87 | Propazine | 78.77 | 84.83 | 2.46 | 0.11 | 91.94 | 92.58 | 10.66 | 0.43 | 89.80 | 107.34 | 6.40 | 0.20 | 0.93 | 0.01 | 19.53 | 0.0014 | 13.59 |
| 88 | Propiconazole | 87.66 | 83.24 | 7.07 | 0.31 | 101.22 | 93.15 | 2.83 | 0.11 | 86.03 | 83.59 | 6.88 | 0.22 | 12.93 | 0.01 | -2.96 | 0.0022 | 9.09 |
| 89 | Propoxur | 75.46 | 82.08 | 10.78 | 0.48 | 87.43 | 91.35 | 2.64 | 0.11 | 90.67 | 104.14 | 5.63 | 0.18 | 5.01 | 0.01 | 14.86 | 0.0018 | 7.66 |
| 90 | Pyraclostrobin | 80.21 | 79.01 | 3.58 | 0.16 | 94.36 | 86.52 | 4.06 | 0.16 | 88.47 | 93.15 | 2.89 | 0.09 | 0.53 | 0.01 | 5.29 | 0.0018 | 7.84 |
| 91 | Pyrazosulfuron-ethyl | 81.78 | 83.34 | 10.50 | 0.47 | 86.86 | 87.27 | 12.06 | 0.48 | 91.08 | 102.31 | 8.93 | 0.28 | 1.27 | 0.01 | 12.33 | 0.0016 | 11.87 |
| 92 | Pyriproxyfen | 84.75 | 74.68 | 5.46 | 0.24 | 62.12 | 51.25 | 12.84 | 0.52 | - | - | - | - | 2.91 | 0.1 | -11.88 | 0.0159 | 9.86 |
| 93 | Quinalphos | 87.09 | 82.93 | 3.71 | 0.16 | 100.27 | 90.81 | 5.80 | 0.23 | 87.55 | 94.43 | 4.89 | 0.15 | 12.72 | 0.01 | 7.86 | 0.0022 | 8.41 |
| 94 | Quizalofop ethyl | 55.72 | 60.07 | 5.04 | 0.22 | - | - | - | - | - | - | - | - | 0.03 | 1 | - | 0.0676 | 6.76 |
| 95 | Simazine | 77.36 | 84.86 | 7.02 | 0.31 | 92.40 | 92.65 | 4.00 | 0.16 | - | - | - | - | 3.08 | 0.05 | 0.27 | 0.0034 | 7.44 |
| 96 | Tebuconazole | 82.81 | 84.89 | 2.63 | 0.12 | 94.40 | 86.25 | 8.01 | 0.32 | 109.36 | 105.91 | 18.46 | 0.58 | 5.07 | 0.01 | -3.15 | 0.0023 | 21.57 |
| 97 | Temephos | 87.60 | 80.96 | 6.29 | 0.28 | 97.99 | 82.10 | 2.97 | 0.12 | - | - | - | - | 1.82 | 0.05 | -16.22 | 0.0089 | 8.01 |
| 98 | Tetramethrin | 84.22 | 77.44 | 5.49 | 0.24 | 99.01 | 88.00 | 6.02 | 0.24 | - | - | - | - | 23.80 | 0.05 | -11.12 | 0.0085 | 8.66 |
| 99 | Thiacloprid | 79.04 | 88.77 | 6.24 | 0.28 | 87.56 | 92.66 | 15.22 | 0.61 | 71.88 | 85.69 | 17.97 | 0.57 | 0.00 | 0.01 | 19.22 | 0.0027 | 20.89 |
| 100 | Thiamethoxam | 81.13 | 85.08 | 9.80 | 0.44 | 110.58 | 106.28 | 17.35 | 0.70 | 88.91 | 118.89 | 9.62 | 0.30 | 1.28 | 0.01 | 33.72 | 0.0023 | 13.85 |
| 101 | Triasulfuron | 70.38 | 80.55 | 4.44 | 0.20 | 75.56 | 82.40 | 15.07 | 0.61 | 70.44 | 119.77 | 14.26 | 0.45 | 0.00 | 0.01 | 70.03 | 0.0024 | 17.40 |
| 102 | Tricyclazole | 71.08 | 82.89 | 2.96 | 0.13 | 102.93 | 119.70 | 11.74 | 0.47 | 83.88 | 91.82 | 12.24 | 0.39 | 3.88 | 0.01 | 9.47 | 0.0018 | 14.98 |
| 103 | Triadimefon | 87.84 | 83.27 | 3.54 | 0.15 | 99.04 | 87.28 | 10.85 | 0.44 | 83.76 | 97.20 | 8.84 | 0.28 | 2.77 | 0.01 | 16.04 | 0.0025 | 10.60 |
|  | Pesticide recovered at |  |  |  |  |  |  |  |  |  |  |  |  |  |  |  |  |  |
|  | <70% | 6 | 7 |  |  | 6 | 7 |  |  | 9 | 2 |  |  |  |  |  |  |  |
|  | **70%-120%** | **95** | **94** |  |  | **91** | **90** |  |  | **77** | **84** |  |  |  |  |  |  |  |
|  | >120% | 1 | 1 |  |  | 1 | 1 |  |  | 0 | 0 |  |  |  |  |  |  |  |
|  | Not detected | 1 | 1 |  |  | 5 | 5 |  |  | 17 | 17 |  |  |  |  |  |  |  |

RSS* = Percent Recovery of Solvent Standard, RMM# = Percent Recovery of Matrix matched Standard, RSD **©=** Percentage Relative Standard Deviation, HorRat●= Horwitz Ratio, Specificity = Specificity of the target pesticides in presence of blank matrix calculated against fortified blank sample at LOQ, ME$ = Matrix effect at LOQ (%), LOQ^&^ = Method Limit of Quantification, GU = Global Uncertainty at LOQ, U (%) = Uncertainty percentage.

Supplementary figures for the manuscript:

**
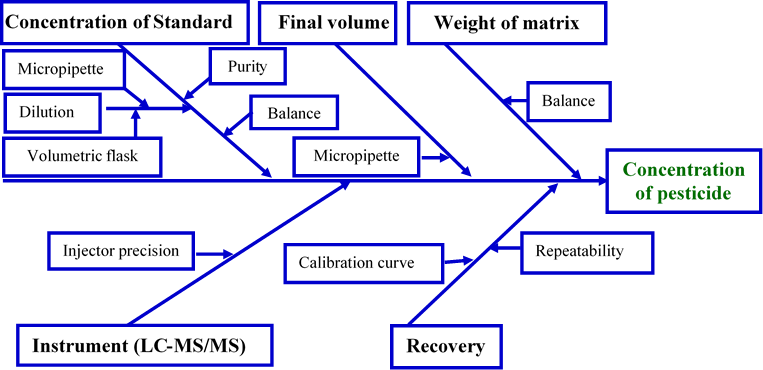
**

**Figure: S1. Fish bone diagram of potential sources of uncertainty in measurement of pesticides in mango fruit drink matrix**

**
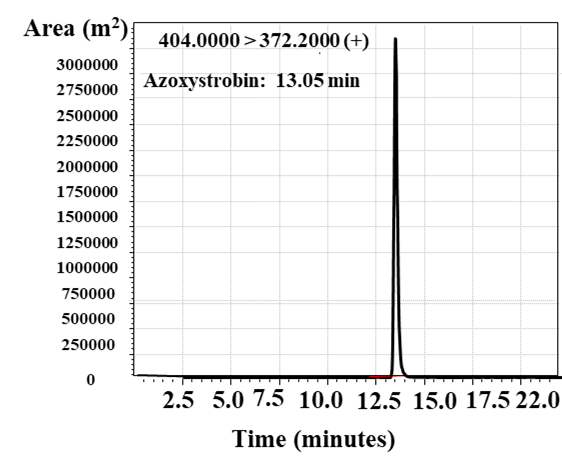
**

**Figure: S2. Specificity for azoxystrobin**

**
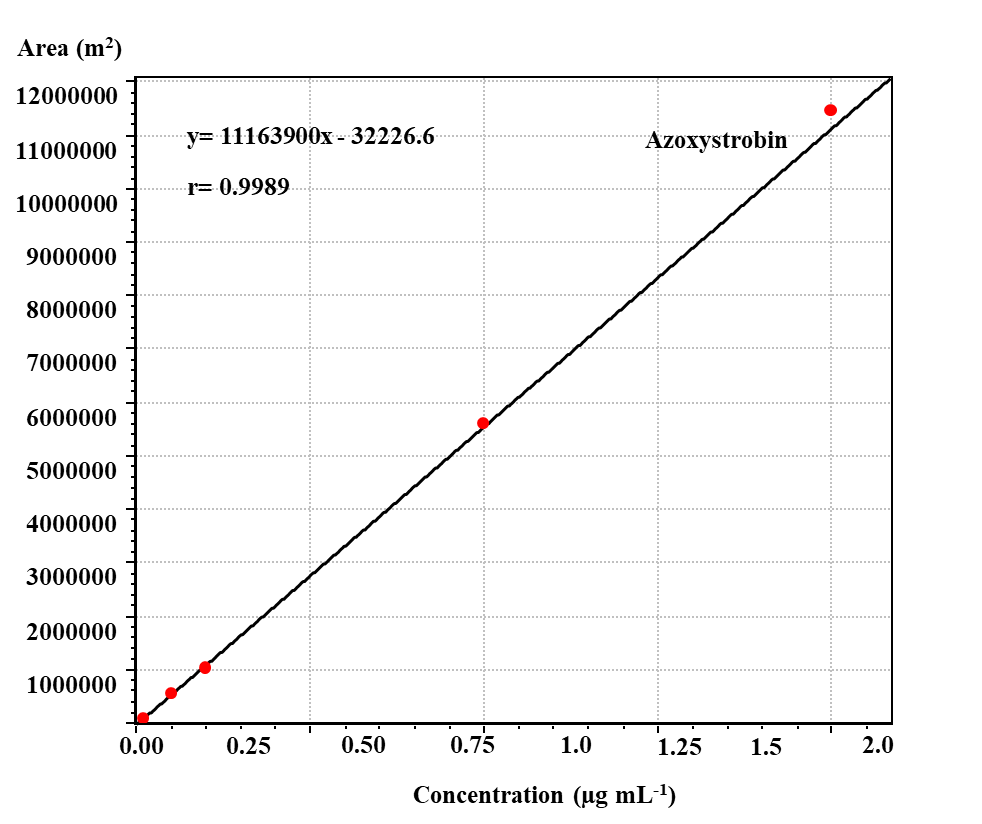
**

**Figure: S3. Linearity curve for azoxystrobin**
